# The Cellular and Synaptic Architecture of the Mechanosensory Dorsal Horn

**DOI:** 10.1016/j.cell.2016.12.010

**Published:** 2017-01-12

**Authors:** Victoria E. Abraira, Emily D. Kuehn, Anda M. Chirila, Mark W. Springel, Alexis A. Toliver, Amanda L. Zimmerman, Lauren L. Orefice, Kieran A. Boyle, Ling Bai, Bryan J. Song, Karleena A. Bashista, Thomas G. O'Neill, Justin Zhuo, Connie Tsan, Jessica Hoynoski, Michael Rutlin, Laura Kus, Vera Niederkofler, Masahiko Watanabe, Susan M. Dymecki, Sacha B. Nelson, Nathaniel Heintz, David I. Hughes, David D. Ginty

**Affiliations:** 1 Department of Neurobiology, Howard Hughes Medical Institute, Harvard Medical School, 220 Longwood Avenue, Boston, MA 02115, USA; 2 Neuroscience Training Program, The Johns Hopkins University School of Medicine, Baltimore, MD 21205, USA; 3 Institute of Neuroscience and Psychology, University of Glasgow, Glasgow G12 8QQ, UK; 4 Department of Biology and National Center for Behavioral Genomics, Brandeis University, Waltham, MA 02453, USA; 5 The Laboratory of Molecular Biology, Howard Hughes Medical Institute, The Rockefeller University, New York, NY 10065, USA; 6 Department of Genetics, Harvard Medical School, 77 Avenue Louis Pasteur, Boston, MA 02115, USA; 7 Department of Anatomy, Hokkaido University School of Medicine, Sapporo 060-8638, Japan

**Keywords:** spinal cord interneurons, low-threshold mechanoreceptors, somatosensation, spinal cord dorsal horn, mouse molecular genetics, synaptic connectivity

## Abstract

The deep dorsal horn is a poorly characterized spinal cord region implicated in processing low-threshold mechanoreceptor (LTMR) information. We report an array of mouse genetic tools for defining neuronal components and functions of the dorsal horn LTMR-recipient zone (LTMR-RZ), a role for LTMR-RZ processing in tactile perception, and the basic logic of LTMR-RZ organization. We found an unexpectedly high degree of neuronal diversity in the LTMR-RZ: seven excitatory and four inhibitory subtypes of interneurons exhibiting unique morphological, physiological, and synaptic properties. Remarkably, LTMRs form synapses on between four and 11 LTMR-RZ interneuron subtypes, while each LTMR-RZ interneuron subtype samples inputs from at least one to three LTMR classes, as well as spinal cord interneurons and corticospinal neurons. Thus, the LTMR-RZ is a somatosensory processing region endowed with a neuronal complexity that rivals the retina and functions to pattern the activity of ascending touch pathways that underlie tactile perception.

## Highlights

• Convergent LTMR and cortical inputs define the mechanosensory dorsal horn
• A dorsal horn molecular-genetic toolbox defines 11 interneuron subtypes
• Dorsal horn interneurons receive specific patterns of cortical and LTMR inputs
• Dorsal horn interneurons modulate output pathways and tactile perception

## Graphical Abstract

**Figure:**
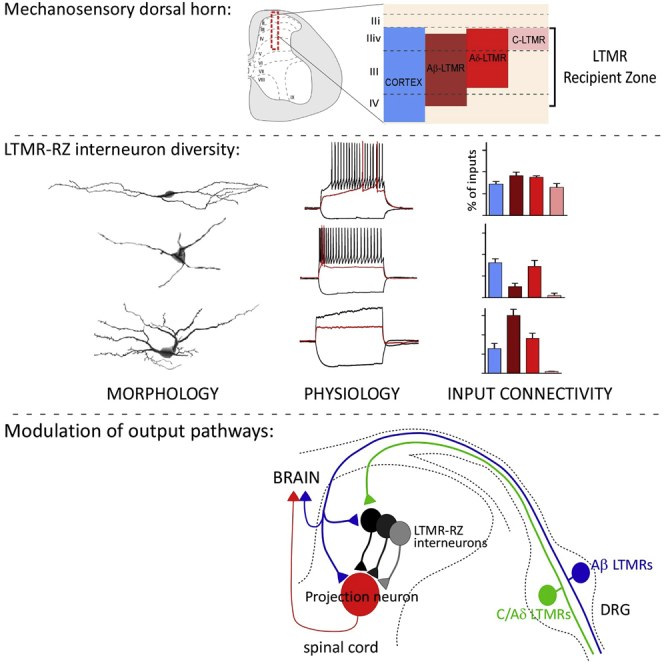

## Teaser

The blueprint of dorsal horn reveals the organizational logic of the low-threshold mechanoreceptor-recipient zone that is essential for tactile perception.

## Introduction

The somatosensory system decodes a wide range of tactile stimuli, thereby endowing us with an extraordinary capacity for object recognition, texture discrimination, and fine motor control. The anatomical substrate of innocuous touch perception is rooted in the intricate innervation patterns of physiologically distinct and morphologically specialized sensory neurons, termed low-threshold mechanoreceptors (LTMRs). The unique morphological and anatomical arrangement of LTMR subtype endings in the skin, our largest sensory organ, underlies distinct LTMR subtype response properties for the perception of object size, shape, texture, vibration, and direction of stimulus movement (Owens and Lumpkin, 2014, Zimmerman et al., 2014). LTMRs also confer complex social and emotional, or affective qualities of touch (Olausson et al., 2002).

Cutaneous LTMR subtypes are classified as Aβ, Aδ, or C based on their action potential conduction velocity (Horch et al., 1977). LTMRs are further distinguished by their preferred stimuli, the cutaneous end organs with which they associate, and by their rates of adaptation to constant indentation of the skin. In mouse hairy skin, guard hair follicles are associated with Aβ RA-LTMRs, Aβ SAI-LTMRs, and Aβ Field-LTMRs, which are differentially sensitive to hair deflection, skin indentation, and stroke and exhibit different rates of adaptation (Abraira and Ginty, 2013, Burgess et al., 1968). Conversely, Awl/Auchene and zigzag hair follicles, which together account for ∼99% of hair follicles across the body, are quadruply innervated by Aβ RA-LTMRs, Aβ Field-LTMRs, Aδ-LTMRs, and C-LTMRs and triply innervated by Aβ Field-LTMRs, Aδ-LTMRs, and C-LTMRs, respectively (Bai et al., 2015, Li et al., 2011). In contrast to hairy skin, the light touch receptors of glabrous skin include Aβ RA1-LTMRs, Aβ RA2-LTMRs, Aβ SA1-LTMRs, and Aβ SA2-LTMRs (Johnson and Hsiao, 1992). Ensembles of LTMR activities emanating from the skin convey tactile information via central projections to the spinal cord and brainstem. Thus, the perception of diverse tactile stimuli requires robust and precise mechanical stimulus detection by LTMR peripheral endings in the skin and intricate processing capabilities of LTMR activity ensembles by interneurons in the CNS. Defining the cellular and synaptic substrates of touch information processing in the CNS will reveal how LTMR activity ensembles, internal state, and experience are integrated to generate percepts of the physical world.

The historical, canonical view of innocuous, discriminative touch information processing in the CNS has emphasized the “direct dorsal column pathway,” in which Aβ-LTMR axonal branches project directly, via the dorsal column, to the brainstem dorsal column nuclei (DCN) where second-order neurons project to the thalamus, and from there to the somatosensory cortex (Johnson and Hsiao, 1992). In the classic “labeled line” model, LTMR subtype integration and processing begins in the somatosensory cortex, with the spinal cord, DCN and thalamus serving as relay stations. An alternate model posits an integrative somatosensory system in which touch information processing begins at the earliest stages of sensory neuron input to the CNS. In the visual system, for example, we now appreciate the retina as a key locus of visual information processing, with retinal ganglion cells conveying highly processed visual information from the retina to a large number of brain regions (Masland, 2001). In an analogous, emerging view of the somatosensory system, the spinal cord dorsal horn mirrors the retina by playing a key role in processing innocuous touch information delivered from LMTR activity ensembles. In support of this idea, only a subset of LTMRs extends an axonal branch via the dorsal column to the DCN, while all LTMRs exhibit axonal branches that terminate in the dorsal horn, including regions devoid of projection neurons, in a highly somatotopic manner (Li et al., 2011). Thus, “indirect,” or post-synaptic, ascending pathways are likely to convey processed and perceptually relevant innocuous touch information from the dorsal horn to the brain. However, the neural substrates and mechanisms of LTMR ensemble integration and processing in the dorsal horn, and the functions of dorsal horn LTMR-recipient zone (LTMR-RZ) interneurons and post-synaptic ascending pathways in touch perception are poorly understood.

In the present study, we sought to define the organizational logic of the spinal cord LTMR-RZ and its role in innocuous touch information processing and tactile perception. Through an open-ended screen to identify genes that are uniquely expressed in select LTMR-RZ neuronal subtypes, and exploitation of these genes for the generation of an array of mouse molecular-genetic tools, we found within the LTMR-RZ seven excitatory and four inhibitory interneuron subtypes, each displaying a unique combination of morphological and physiological properties. Moreover, the generation of an excitatory synaptic atlas of the LTMR-RZ revealed that LTMR subtypes form synapses onto four to 11 LTMR-RZ interneuron subtypes. Each of the 11 LTMR-RZ interneuron subtypes receives convergent synaptic inputs from at least one to three LTMR subtypes, as well as other locally projecting LTMR-RZ interneurons and corticospinal projection neurons. We also found that LTMR-RZ interneurons play essential roles in innocuous touch perception and tune the responses of postsynaptic ascending projection pathway neurons that convey touch information from the spinal cord to the brain. Thus, the LTMR-RZ is a complex and highly interconnected locus of LTMR and cortical input integration that orchestrates the activity of postsynaptic ascending pathways required for innocuous touch perception.

## Results

### The Mechanosensory Dorsal Horn Is Defined by Overlapping LTMR and Cortical Inputs and Comprises a Large Diversity of Locally Projecting Interneurons

**Figure 1:**
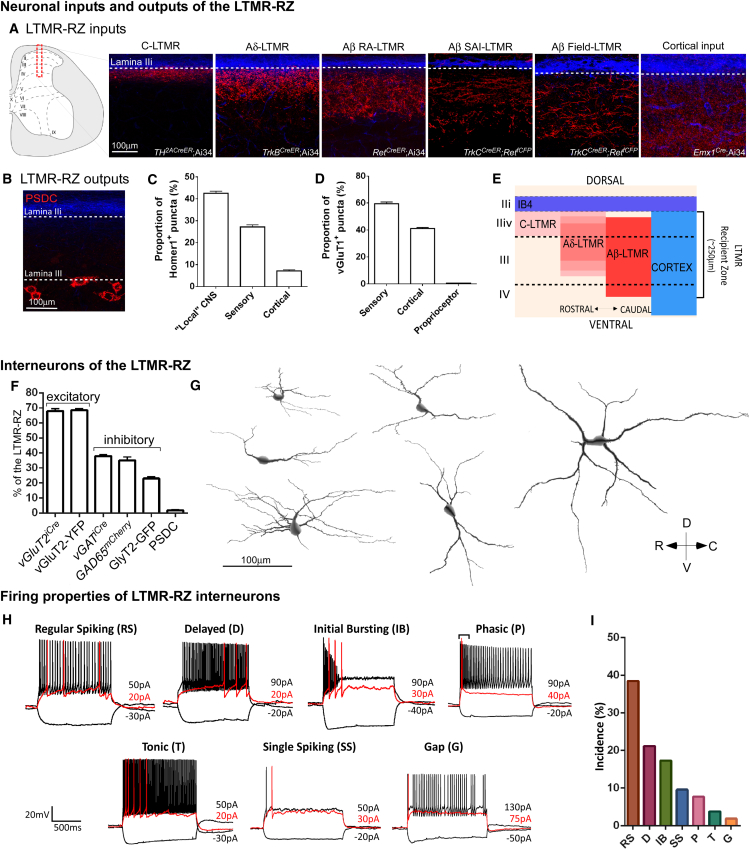
The Mechanosensory Dorsal Horn Is Defined by Overlapping LTMR and Cortical Inputs and Comprises a Large Diversity of Locally Projecting Interneurons (A) Sagittal sections of adult mouse lumbar spinal cord dorsal horn at the level shown in the schematic (left) depicting inputs from all genetically defined classes of LTMRs, as well as cortical input. IB4 binding in blue labels lamina IIi. (B) Sagittal section of adult mouse spinal cord with post-synaptic dorsal column neurons (PSDCs) labeled in red. IB4 is labeled in blue. (C) Percentage of Homer1^+^ puncta within the LTMR-RZ opposed to synaptic inputs originating in the spinal cord, dorsal root ganglia, and cortex. (D) Percentage of vGluT1^+^ terminals within the LTMR-RZ that overlap with sensory, cortical, and proprioceptive inputs. (E) Schematic summarizing input modalities and anatomical depth of the LTMR-RZ. (F) Percentage of LTMR-RZ neurons that are excitatory, inhibitory, or projections neurons. (G) Sample Neurolucida reconstructions of LTMR-RZ interneurons labeled randomly. (H) Sample action potential discharge patterns of randomly recorded LTMR-RZ interneurons during somatic injection of hyperpolarizing and depolarizing current steps of increasing magnitude (black traces, rheobase trace in red, current step magnitude noted in pA). Bracket over phasic trace denotes the burst of action potentials (APs) at rheobase distinctive of this particular discharge pattern (n = 52). (I) Percentage of incidence of the seven LTMR-RZ interneuron firing properties depicted in (H). For further details on genetic crosses, see STAR Methods. See also Figure S1.

**Figure S1:**
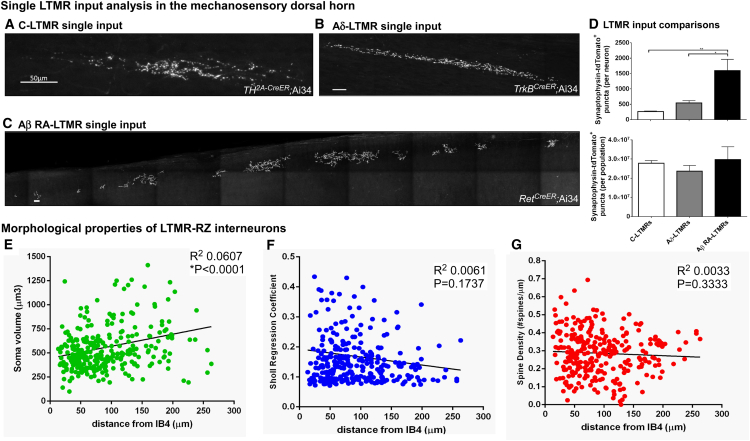
Additional Characterization of the LTMR-RZ, Related to Figure 1 (A) Whole mount labeling of a single C-LTMR input with *TH*^*2A-CreER*^;*R26*^*LSL-synaptophysin-tdTomato*^(Ai34) and 0.02mg of tamoxifen at P21. (B) Whole mount labeling of a single Aδ-LTMR input with *TrkB*^*CreER*^;Ai34 and 0.25mg of tamoxifen at P21. (C) Whole mount labeling of a single Aβ RA-LTMR input with *Ret*^*CreER*^;Ai34 and 0.02mg of tamoxifen at E10.5. (D) LTMR single input comparisons. Top panel shows average number of synapses per neuron (n = 4 for each LTMR subtype). Published data citing an average of 10,000 neurons per mouse DRG (Gjerstad et al., 2002), and relative proportions of DRG neurons that comprise the C-, Aδ-, and Aβ RA-LTMR populations as 15%–20%, 7%, and 5%, respectively (Li et al., 2011, Rutlin et al., 2015, Luo et al., 2009) was used to subsequently calculate the approximate number of total synapses from each population (lower panel, see STAR Methods). For puncta per neuron: (one-way ANOVA: p = 0.0039, F(2,9) = 10.96). Post hoc Tukey’s test: ^∗^p < 0.05, ^∗∗^p < 0.01. (E) Plot of soma volume as a function of distance from IB4 (Lamina IIiv/III boundary). (F) Plot of Sholl Regression Coefficient (k) as a function of distance from IB4. Sholl Regression Coefficient (k) is a Sholl-based measure that describes the change in dendrite density as a function of distance from the cell body. A low k value is often associated with a high neurite complexity. These results show that both simple and complex neurite morphologies can be found throughout the LTMR-RZ. (G) Plot of spine density as a function of distance from IB4. Spine density measurements can be an indicator of excitatory and inhibitory subtypes, with inhibitory neurons often having very low spine density counts. These results suggest that both excitatory and inhibitory interneurons can be found throughout the LTMR-RZ.

We localized initial sites of innocuous touch information processing by visualizing LTMR subtype endings in the spinal cord dorsal horn. The organization of synaptic inputs of C-LTMRs, Aδ-LTMRs, Aβ RA-LTMRs, Aβ SAI-LTMRs, and Aβ Field-LTMRs in the mouse dorsal horn was assessed using LTMR-CreER and intersectional mouse genetic tools (Figure 1A; Bai et al., 2015, Li et al., 2011, Luo et al., 2009, Rutlin et al., 2015). This analysis showed that LTMR inputs to the dorsal horn are organized in a highly overlapping fashion spanning ∼250 μm below IB4^+^ lamina IIi, in a region of the dorsal horn, which we have termed the LTMR-Recipient Zone (LTMR-RZ, Figure 1E). We estimate that the total number of C-LTMR, Aδ-LTMR, and individual Aβ-LTMR subtype synapses within the LTMR-RZ are comparable (Figures S1A–S1D), suggesting equal synaptic allocation of LTMR subtypes within the dorsal horn. The most prominent ascending pathway emanating from the LTMR-RZ is the post-synaptic dorsal column (PSDC) pathway (Rustioni and Kaufman, 1977) visualized by retrograde labeling from the dorsal columns and found to be located at the lamina III/IV boundary of the LTMR-RZ (Figure 1B). Interestingly, sensory neurons and locally projecting interneurons together account for only ∼70% of total glutamatergic excitatory inputs to the LTMR-RZ (Figure 1C). Thus, we next sought to uncover additional synaptic inputs that contribute to the excitatory drive in the LTMR-RZ.

In other sensory systems, cortical activity plays a crucial role in sensory processing (Otazu et al., 2015), and a large fraction of corticospinal neurons originate in the somatosensory cortex and preferentially innervate the dorsal horn of species ranging from rodents to primates (Casale et al., 1988, Ralston and Ralston, 1985). Labeling of cortical projection neurons in mice using *Emx1*^*Cre*^ revealed that cortical neuron synapses account for ∼40% of vGluT1^+^ synapses in the LTMR-RZ, which together with primary somatosensory terminals labeled with *Advillin*^*Cre*^ accounts for virtually 100% of vGluT1^+^ synapses within this region (Figure 1D). Remarkably, cortical projection neuron synapses and LTMR subtype synapses together sharply define the upper region of the LTMR-RZ (Figures 1A and 1E).

We next sought to define the neuronal substrates of innocuous touch information processing within this spinal cord region. Labeling of excitatory and inhibitory neuronal subtypes revealed that ∼70% of neurons intrinsic to the LTMR-RZ are excitatory while ∼30% are inhibitory (Figure 1F). Retrograde labeling of known supraspinal projecting neurons originating in the LTMR-RZ (PSDC and anterolateral tract neurons, ALT) revealed that these projection neurons represent fewer than 2% of neurons in this region (Figure 1F; S. Choi and D.D.G., unpublished data), and thus, the vast majority of LTMR-RZ neurons project locally, likely within the spinal cord itself.

The extent of LTMR-RZ interneuron subtype diversity was next defined by assessing their morphological and physiological properties, which are largely unexplored. Morphological diversity of LTMR-RZ interneurons was assessed using an unbiased genetic labeling approach (see STAR Methods) to sparsely label, reconstruct, and morphometrically analyze 305 individual neurons. This revealed a broad range of morphological complexity in the LTMR-RZ (Figure 1G), with a correlation that suggests an increase in cell body size as a function of distance ventral to the IB4 layer (Figure S1E). Neurons exhibiting a range of spine densities and branching patterns are spread evenly throughout the LTMR-RZ, indicating an intermingling of excitatory and inhibitory neurons with varied morphologies (Figures S1F and S1G). The extent of physiological diversity of LTMR-RZ neurons was assessed using whole-cell patch-clamp recordings of randomly chosen neurons (n = 52). Current injections into randomly chosen neurons revealed neuronal types exhibiting diverse firing patterns, including single spiking, initial bursting, phasic, delayed, gap, regular spiking, and tonic firing patterns (Figures 1H and 1I), some of which had been observed previously in the rodent superficial dorsal horn (Grudt and Perl, 2002, Yasaka et al., 2010). Taken together, the LTMR-RZ is a complex spinal cord region composed mainly of local interneurons exhibiting a wide range of morphological and physiological properties.

### A Dorsal Horn Molecular-Genetic Toolbox for Excitatory and Inhibitory Interneuron Subtypes of the LTMR-RZ

**Figure 2:**
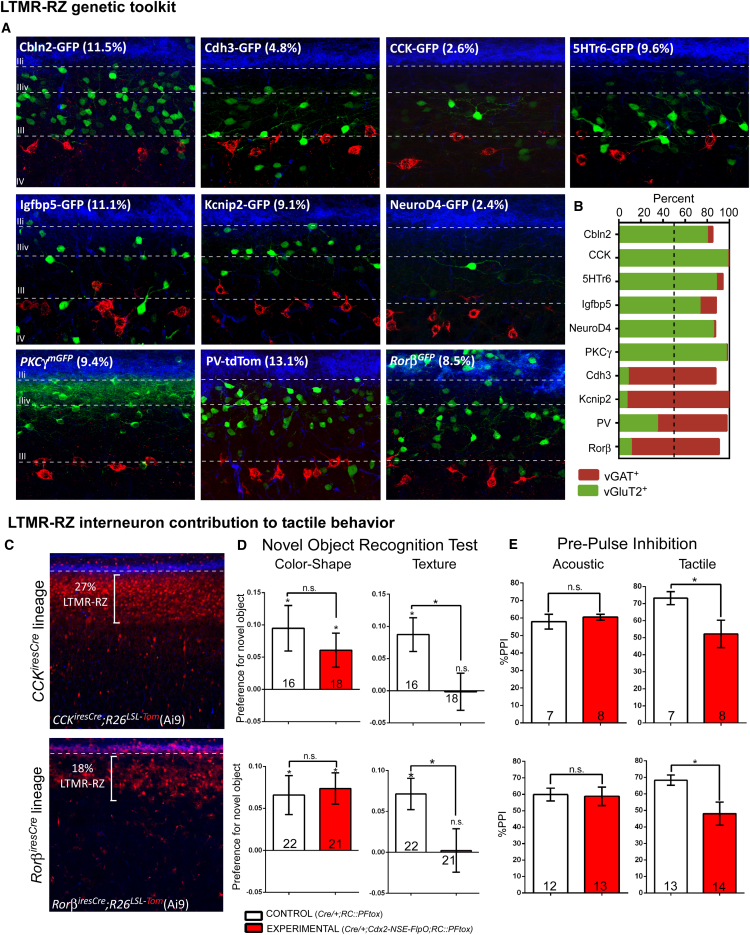
An LTMR-RZ Genetic Toolkit and Contributions of LTMR-RZ Interneurons to Tactile Perception (A) Sagittal sections of the LTMR-RZ from the interneuron GFP/Tomato mouse lines. Fluorescent reporters are in green, CTB-labeled PSDCs are in red, IB4 binding is in blue. Percentage of the LTMR-RZ is in parentheses. (B) Neurotransmitter quantification for the ten interneuron lines. Excitatory and inhibitory neurons labeled with *vGluT2*^*iresCre*^ and *vGAT*^*iresCre*^ mouse lines, respectively. (C) Sagittal spinal cord section from a *CCK*^*iresCre*^;*R26*^*LSL-tdTom*^(Ai9) mouse and an *Rorβ*^*iresCre*^;*R26*^*LSL-TdTom*^(Ai9) mouse. IB4 lamina IIi in blue. (D) Discrimination indices for color-shape NORT (left) and texture NORT (right). *CCK*^*iresCre*^;Cdx2-NSE-FlpO;*RC*::*PFTox* animals (top), *Rorβ*^*iresCre*^;Cdx2-NSE-FlpO;*RC*::*PFTox* animals (bottom). Positive value indicates preference for the novel object compared to the familiar object. Values displayed as percentages. ^∗^p < 0.05. (E) Percentage of inhibition of startle response to 125 dB noise in control and mutant littermates when the startle noise is preceded by an 80dB acoustic prepulse (left) or a light air puff of 1.5 PSI (right). *CCK*^*iresCre*^;Cdx2-NSE-FlpO*; RC*::*PFTox* animals (top), *Rorβ*^*iresCre*^;Cdx2-NSE-FlpO;*RC*::*PFTox* animals (bottom). ^∗^p < 0.05. For further details and statistical methods used, see STAR Methods. See also Figures S2 and S3; Table S1.

**Figure S2:**
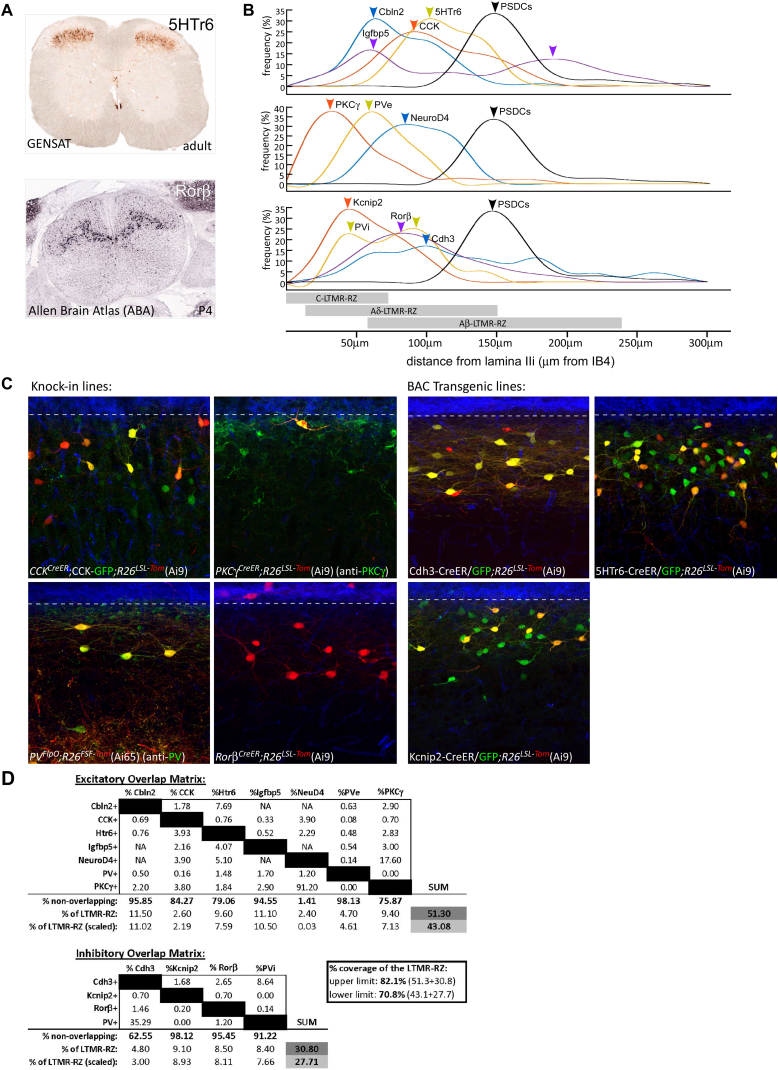
Additional Characterization of LTMR-RZ Genetic Toolbox, Related to Figure 2 (A) Examples of transverse spinal cord images from GENSAT (top, http://www.gensat.org/index.html) and Allen Brain Atlas (bottom, http://mousespinal.brain-map.org) websites depicting expression patterns screened for during *in-silico* screen. (B) Smoothened cell body histogram distribution of LTMR-RZ interneuron lines. Arrows indicate peak frequency of soma location within the LTMR-RZ. (C) Sagittal sections of the LTMR-RZ from CreER/FlpO knockin animals (left) and BAC-transgenic CreER lines (right). IB4 binding in blue. Animal genotype on the bottom left corner. Recombinase activity is depicted in red. Antibody binding, in the case for PKCγ and PV, or overlap with fluorescent reporter lines depicted in green. Also, see Table S1B. (D) Excitatory and inhibitory overlap matrix used to calculate the percent coverage of the LTMR-RZ represented by the eleven genetically labeled interneuron lines. Each box in the matrix represents a unique mouse cross to assess the amount of overlap between the two mouse lines. For each mouse line, the “% non-overlapping” is derived by adding the percent overlap (ie each matrix box in the column) and subtracting it from 100. The “% of the LTMR-RZ” are as depicted in Figure 2A for each individual line, the sum of which represents the coverage of the LTMR-RZ without consideration for potential overlap (51.3%+30.8% = 82.1%). The “% of LTMR-RZ (scaled)” represents the percentage of the LTMR-RZ that each line represents scaled for the overlapping population. The sum of this scaled percentage represents the coverage of the LTMR-RZ taking into consideration the amount of overlap across each mouse line (43.1%+27.7% = 70.8%). See STAR Methods for mouse crosses, at least 100 GFP^+^ neurons counted per animal, at least 3 animals per cross. Percent overlap with PVe and PVi is calculated as 36% excitatory and 64% inhibitory. NA: mouse lines not available for compatible crosses.

We next sought to establish mouse molecular-genetic tools useful for defining the properties, organization, and function of the morphologically and physiologically diverse LTMR-RZ interneuron populations. We conducted in silico screens of publicly available atlases: Gene Expression Nervous System Atlas and Allen Brain Atlas, for genes exhibiting expression within the adult mouse LTMR-RZ, but not the intermediate or ventral spinal cord (Figure S2A).This screen culminated in the characterization and/or production of ten fluorescent reporter BAC transgenic or knockin mouse lines that label morphologically homogeneous subsets of LTMR-RZ interneurons. Each of these lines labels 13% or fewer of all LTMR-RZ neurons (Figure 2A). The LTMR-RZ mouse lines include GENSAT BAC-GFP transgenic lines for genes that encode the cell adhesion molecules Cadherin-3 (Cdh3) and Cerebellin-2 (Cbln2), the neuropeptide cholecystokinin (CCK), Serotonin Receptor 6 (5HTr6), Insulin-like Growth Factor Binding Protein 5 (Igfbp5), Kv Channel Interacting protein-2 (Kcnip2), Neurogenic Differentiation Factor-4 (NeuroD4), and a PV-tdTomato BAC transgenic line (Kaiser et al., 2016). We also generated or obtained *PKCγ*^*mGFP*^ and *Rorβ*^*GFP*^ knockin lines, which label the PKCγ^+^ and Rorβ^+^ interneuron populations, respectively (Table S1A; Liu et al., 2013).

The extent to which the ten genetically labeled interneuron lines represent unique subsets of excitatory or inhibitory neurons within the LTMR-RZ was next determined. For this, each of the ten fluorescent reporter lines (Figure 2A) was crossed with mice in which excitatory and inhibitory interneurons were labeled using either *vGluT2*^*iresCre*^ or *vGAT*^*iresCre*^ and Cre-dependent reporters. This analysis revealed that six of the ten fluorescent reporter lines predominantly label excitatory neurons, while three lines label inhibitory interneurons (Figure 2B). Similar to the rat dorsal horn, we found that approximately 70% of PV^+^ interneurons in laminae I–III contain GABA and glycine (Hughes et al., 2012, Laing et al., 1994). Therefore, the PV^+^ neuronal population was subdivided into PVe and PVi subtypes, thus yielding a total of ten genes that label 11 putative neuronal subtypes. Anatomical distribution analysis of these 11 subtypes showed that each is broadly distributed throughout the LTMR-RZ, with a subset being more prominently localized to particular lamina (Figure S2B).

We next sought to increase the versatility of the LTMR-RZ interneuron genetic toolbox by generating or acquiring recombinase tools for the majority of the ten genes that label LTMR-RZ neuronal subsets. We generated Cdh3-CreER, 5HTr6-CreER, and Kcnip2-CreER BAC transgenic lines as well as *PKCγ*^*CreER*^ and *Rorβ*^*CreER*^ knockin mouse lines and acquired the previously reported *CCK*^*CreER*^ and *PV*^*FlpO*^ knockin mouse lines (Figure S2C; Table S1B; Taniguchi et al., 2011). In combination with fluorescent reporter lines and antibodies for immunohistochemistry, these recombinase lines enabled an assessment of the extent of overlap between the 11 LTMR-RZ interneuron populations; the fluorescent reporter lines were found to represent largely non-overlapping populations within the excitatory and inhibitory cohorts, with some minor exceptions (Figure S2D). Taking into consideration the percentage of coverage of each fluorescent reporter line, as well as the excitatory/inhibitory matrix analysis, the overlap measurements revealed that the fluorescent reporter lines together account for between 71% and 82% coverage of all LTMR-RZ neurons (Figure S2D).

### LTMR-RZ Interneurons Contribute to Tactile Perception

**Figure S3:**
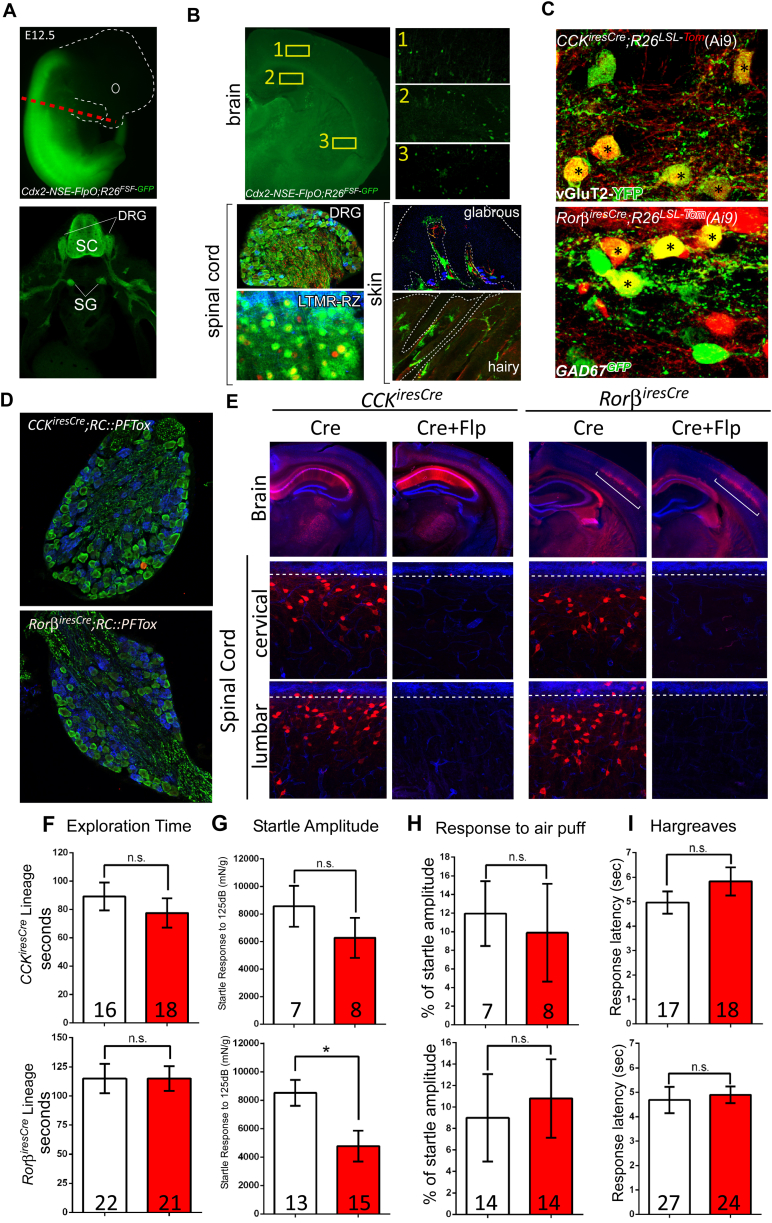
Characterization of Intersectional Inactivation and Additional Behavioral Assays, Related to Figure 2 (A) Cdx2-NSE-FlpO;*R26*^*FSF-GFP*^ E12.5 embryo depicting caudal expression of FlpO (top). Cross section at red dotted line (bottom). Early in development Cdx2-NSE-FlpO recombination is restricted to posterior neural plate, prospective spinal cord territory. See STAR Methods and (Coutaud and Pilon 2013). Note specific FlpO expression in caudal neuronal tissues (spinal cord, SC; dorsal root ganglia, DRG; sympathetic ganglia, SG) but not in brain, internal organs or skin. (B) Adult characterization of brain, spinal cord, and skin tissue from a Cdx2-NSE-FlpO; *R26*^*FSF-GFP*^ animal. Adult brain characterization reveals very sparse FlpO activity in the brain (top). Yellow insets show very low levels of recombination in the cortex (1), hippocampus (2), and striatum (3). Adult DRG and spinal tissue show near complete FlpO recombination (bottom left, IB4 binding in blue). Adult glabrous and hairy skin sections (bottom right) show no FlpO activity in skin cells (outlined in white dotted lines) including Troma1+ merkel cells depicted in blue for the glabrous skin inset. Neurofilament 200 staining in red, GFP staining in green. (C) Neurotransmitter characterization of *CCK*^*iresCre*^ and *Rorβ*^*iresCre*^ lineages in the LTMR-RZ. Asterisk denotes overlap. (D) DRG cross-sections from *CCK*^*iresCre*^*;RC::PFtox* (top) and *Rorβ*^*iresCre*^*;RC::PFtox* (bottom) animals. *Cre* recombination of *RC::PFtox* results in *mCherry* expression, depicted in red. Note very minimal DRG Cre recombination of *CCK*^*iresCre*^ (top) and no DRG Cre recombination of *Rorβ*^*iresCre*^ (bottom). IB4 binding in blue, Neurofilament-200 staining in green. (E) Cross-sections through brain and cervical/lumbar spinal cords from *CCK*^*iresCre*^;*RC*::*PFtox*, *CCK*^*iresCre*^;Cdx2-NSE-FlpO;*RC*::*PFtox*, *Rorβ*^*iresCre*^;*RC*::*PFtox and Rorβ*^*iresCre*^;Cdx2-NSE-FlpO;*RC*::*PFtox* animals (left to right). Cre recombination of *RC*::*PFtox* results in mCherry expression in brain and spinal cord, depicted in red. Combined Cre and Flp recombination from Cdx2-NSE-FlpO of *RC*::*PFtox* results in loss of mCherry expression and expression of Tetanus Toxin specifically in spinal cord but not in the brain. For brain sections NeuN is depicted in blue, for spinal cord sections IB4 binding is depicted in blue. (F–H) Additional behavior assays *CCK*^*iresCre*^;Cdx2-NSE-FlpO;*RC*::*PFtox* (top panels), *Rorβ*^*iresCre*^;Cdx2-NSE-FlpO;*RC*:;*PFtox* (bottom panels). (F) Exploration time during texture NORT. (G) Startle amplitude to 125dB noise during PPI test. *Rorβ*^*iresCre*^;Cdx2-NSE-FlpO;*RC*::*PFtox* mutant animals display a much lower startle response than control littermates, indicating some motor deficits (^∗^p < 0.05). (H) Response to a light air puff stimulus alone. Responses are expressed as a percent of startle response to a 125-dB noise. (I) Hargreaves temperature sensitivity assay.

Our characterization of recombinase tools that label LTMR-RZ interneuron subtypes also resulted in the identification of “large lineage” genetic tools, including *CCK*^*iresCre*^ and *Rorβ*^*iresCre*^, which label 27% and 18% of LTMR-RZ interneurons, respectively (Figure 2C; Taniguchi et al., 2011). Neurotransmitter characterization of these lineages revealed that *CCK*^*iresCre*^-labeled neurons are 92% excitatory, while *Rorβ*^*iresCre*^-labeled neurons are 62% inhibitory (Figure S3C). Thus, *CCK*^*iresCre*^ and *Rorβ*^*iresCre*^ are useful for functionally manipulating large cohorts of excitatory and inhibitory LTMR-RZ interneurons, allowing us to ask whether LTMR-RZ interneurons contribute to tactile perception. In order to restrict neuronal manipulations to the spinal cord, as most of the genes identified are expressed in supraspinal centers and also in non-neuronal tissues, we developed an intersectional genetic strategy by generating a neural specific enhancer Cdx2-FlpO mouse line (Cdx2-NSE-FlpO, (Coutaud and Pilon, 2013) that expresses FlpO in the spinal cord, but not in the brain, skin, or internal organs (Figures S3A and S3B). Thus, intersectional inactivation of large LTMR-RZ lineages using either *CCK*^*iresCre*^ and *Rorβ*^*iresCre*^ in conjunction with Cdx2-NSE-FlpO and the dual recombinase tetanus toxin mouse line *RC*:*PFtox* (Niederkofler et al., 2016) was done to assess the role of LTMR-RZ interneurons in tactile perception (Figures S3D and S3E).

To evaluate texture discrimination abilities in mice, we used a texture-specific novel object recognition test (NORT, Orefice et al., 2016). As previously observed, control mice preferentially explored a cube with a novel texture, indicating an ability to discriminate between the familiar and novel textured objects and hence, perception of textured surfaces. In contrast, mice in which either *CCK*^*iresCre*^ or *Rorβ*^*iresCre*^-labeled interneuron lineages were silenced using the intersectional genetic strategy did not show a preference for the novel textured object in this assay (Figures 2D and S3F). Novelty-seeking behavior is not impaired in the mutant animals, as both control and mutant mice showed a significant preference for novel objects that differ in color and shape (Figure 2D). We also asked whether LTMR-RZ neurons contribute to hairy skin sensitivity using a tactile prepulse inhibition of the startle reflex assay (tactile PPI), in which a light air puff prepulse (1.5 PSI) is applied to back hairy skin followed by a startle pulse of broadband white noise (125 dB) to elicit an acoustic startle response (Orefice et al., 2016). As expected, a light air puff prepulse reduced the magnitude of the acoustic startle response in control animals (Figure 2E). However, mutant mice in which either *CCK*^*iresCre*^ or *Rorβ*^*iresCre*^ lineages were silenced exhibited a reduction in tactile PPI performance (Figure 2E). This deficit is specific to tactile responses, as both control and mutant littermates performed comparably in an acoustic version of PPI, where the prepulse is a non-startling broadband white noise of 80 dB (Figure 2E). Aside from these texture discrimination and hairy skin sensitivity defects, both mutant lines exhibited normal gross locomotive behaviors as well as responses to temperature (Figure S3I). Thus, excitatory and inhibitory LTMR-RZ interneuron subtypes are required for texture discrimination and normal hairy skin tactile sensitivity, implicating LTMR-RZ interneurons as critical for innocuous touch perception.

### LTMR-RZ Interneuron Subtypes Exhibit Distinctive Physiological and Morphological Properties

**Figure 3:**
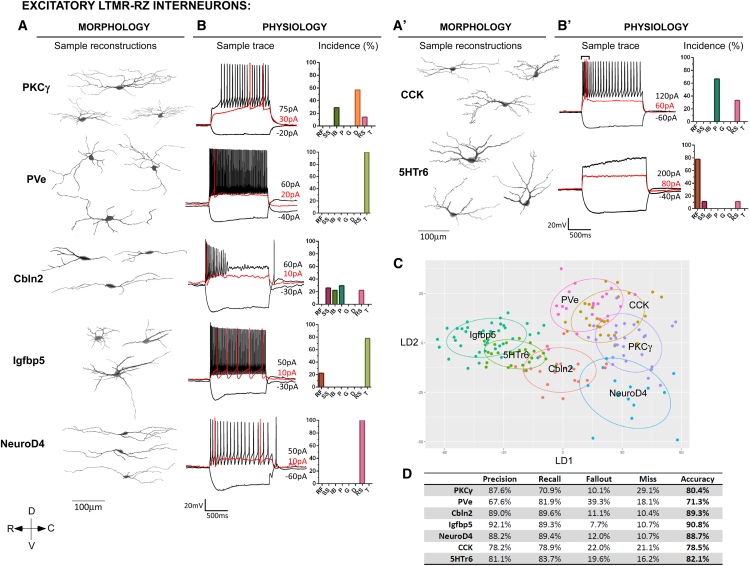
Morphological and Physiological Characterization of Excitatory LTMR-RZ Interneurons (A and A′) Sample Neurolucida reconstructions from the seven excitatory LTMR-RZ interneuron lines. (B and B′) Sample action potential discharge patterns (left) during somatic injection of hyperpolarizing and depolarizing current steps of increasing magnitude (black traces, rheobase trace in red, current step magnitude noted in pA). Percentage of quantification of firing properties (right). (C) Representative plot of an excitatory interneuron training set chosen at random for linear discriminant analysis, demonstrating grouping of excitatory interneuron classes when described by the first two linear discriminants. Ellipses demarcate significant 95% confidence intervals for each interneuron subtype. (D) Performance of an excitatory interneuron classifier generated using linear discriminant analysis. Classifier predictive performance is quantified by precision (positive predictive value), recall (true positive value), fallout (false positive rate), miss (false negative rate), and accuracy (true positive and true negative rate). For further details, see STAR Methods. See also Figure S4.

**Figure 4:**
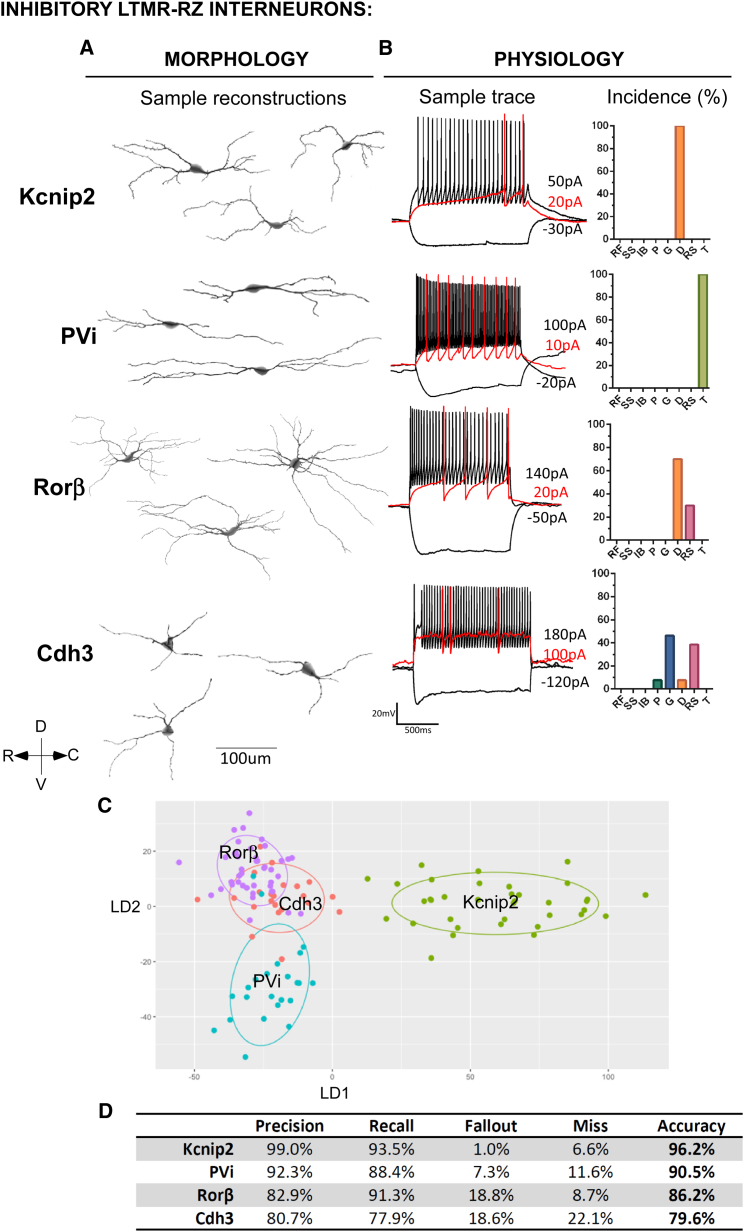
Morphological and Physiological Characterization Inhibitory LTMR-RZ Interneurons (A) Sample Neurolucida reconstructions from the four inhibitory LTMR-RZ interneuron lines. (B) Sample action potential discharge patterns (left) during somatic injection of hyperpolarizing and depolarizing current steps of increasing magnitude. Percentage of quantification of firing properties (right). (C and D) See legend for Figures 3C and 3D. For further details, see STAR Methods. See also Figure S4.

**Figure S4:**
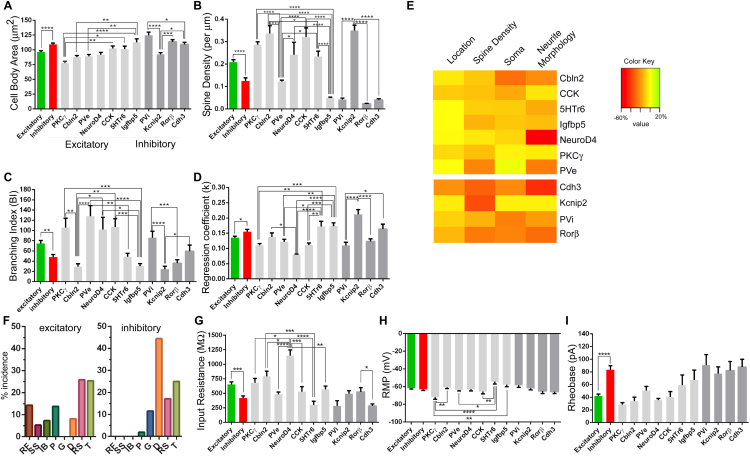
Additional Morphometric and Physiological Characterization of 11 Interneurons of the LTMR-RZ, Related to Figures 3 and 4 (A) Cell body area summary for excitatory and inhibitory subtypes. For excitatory versus inhibitory comparison: (unpaired t test ^∗∗∗∗^p < 0.0001). For excitatory group: (one-way ANOVA: p < 0.0001, F[6,201] = 6.562). For inhibitory group: (one-way ANOVA: p < 0.0001, F[3,142] = 12.47). Post hoc Tukey’s test: ^∗^p < 0.05, ^∗∗^p < 0.01, ^∗∗∗^p < 0.0005, ^∗∗∗∗^p < 0.0001. (B) Spine density measurements for excitatory and inhibitory subtypes. For excitatory versus inhibitory comparison: (unpaired t test ^∗∗^p < 0.0001). For excitatory group: (one-way ANOVA: p < 0.0001, F[6,187] = 24.39). For inhibitory group: (one-way ANOVA: p < 0.0001, F[3,125] = 132.1). Post hoc Tukey’s test: ^∗^p < 0.05, ^∗∗∗∗^p < 0.0001. (C) Branching index (BI) summary describing ramification patterns for excitatory and inhibitory subtypes. BI values are positively correlated to branching complexity. For excitatory versus inhibitory comparison: (unpaired t test ^∗∗^p < 0.005). For excitatory group: (one-way ANOVA: p < 0.0001, F[6,194] = 9.207). For inhibitory group: (one-way ANOVA: p < 0.0001, F[3,138] = 8.952). Post hoc Tukey’s test: ^∗^p < 0.05, ^∗∗^p < 0.005, ^∗∗∗^p < 0.0005, ^∗∗∗∗^p < 0.0001. (D) Regression Coefficient (k) summary for excitatory and inhibitory cohorts describing one sholl-based metric of neurite complexity. k values are negatively correlated to branching complexity. For excitatory versus inhibitory comparison: (unpaired t test ^∗^p < 0.05). For excitatory group: (one-way ANOVA: p < 0.0001, F[6,194] = 9.28). For inhibitory group: (one-way ANOVA: p < 0.0001, F[3,138] = 13.17). Post hoc Tukey’s test: ^∗^p < 0.05, ^∗∗^p < 0.005, ^∗∗∗^p < 0.0005, ^∗∗∗∗^p < 0.0001. (E) Heatmap of changes in classifier accuracy for excitatory and inhibitory interneurons when metrics related to cell location, soma morphology, dendritic spines, or dendrite morphology are omitted from LDA (see STAR Methods for detailed metric membership in each category). Heatmap quantities are displayed as percent change in accuracy (true positive and true negative rate) when one of these categories are omitted, as compared to when all metrics are used to train the linear discriminant model. (F) Percent quantification of action potential discharge patterns for excitatory (left) and inhibitory (right) cohorts. RF = Reluctant Firer, SS = single spiking, IB = Initial Bursting, p = Phasic, G = Gap, D = Delayed, RS = Regular Spiking; T = Tonic. (G) Input Resistance for excitatory and inhibitory subtypes. For excitatory versus inhibitory cohort comparison: (unpaired t test ^∗∗∗^p < 0.0005). For excitatory group: (one-way ANOVA: p < 0.0001, F[6,70] = 9.516). For inhibitory group: (one-way ANOVA: p < 0.05, F[3,39] = 3.950). Post hoc Tukey’s test: ^∗^p < 0.05, ^∗∗^p < 0.005, ^∗∗∗^p < 0.0005, ^∗∗∗∗^p < 0.0001. (H) Resting membrane potential for excitatory and inhibitory subtypes. For excitatory versus inhibitory cohort comparison: (unpaired t test: n.s.). For excitatory group: (one-way ANOVA: p < 0.001, F[6,10] = 5.966). For inhibitory group: (one-way ANOVA: p = 0.1918, F[3,39] = 1.658). Post hoc Tukey’s test: ^∗^p < 0.05, ^∗∗^p < 0.01, ^∗∗∗∗^p < 0.0001. (I) Rheobase currents for excitatory and inhibitory subtypes. For excitatory versus inhibitory cohort comparison: (unpaired t test ^∗∗∗∗^p < 0.0001). For excitatory group: (one-way ANOVA: p = 0.0497, F[6,61] = 2.255). For inhibitory group: (one-way ANOVA: p = 0.9032, F[3,37] = 0.1891).

Our behavioral findings motivated an extensive analysis of the morphological, physiological, and synaptic properties of the 11 genetically labeled interneuron subtypes, and their relationships to ascending projection pathways, to define the nature of LTMR-RZ circuits that underlie touch information processing. For morphological analysis, 351 individual neurons representing each of the 11 genetically labeled subtypes were reconstructed using Neurolucida (Figures 3A and 4A), and 46 parameters that define the morphological features of each neuron were analyzed, including cell body size, neurite length, spine density, and neurite complexity using Sholl-based metrics and Branching Index measurements (Figures S4A–S4D, see STAR Methods). Taken together, this analysis revealed that excitatory LTMR-RZ interneuron subtypes tend to have smaller cell bodies (Figure S4A), more complex neurite morphologies (Figures S4C and S4D) and greater spine densities compared to the inhibitory cell types (Figure S4B). Importantly, linear discriminant analysis (LDA) using the 26 most salient morphological parameters suggested that each genetically labeled interneuron subtype exhibits a unique, distinguishable combination of morphological features (Figures 3C and 4C). These combinations of morphological features were used to create linear classifiers that recognize interneuron subtypes with 83% and 88% accuracy for excitatory and inhibitory interneuron subtypes, respectively (Figures 3D and 4D).

We next asked whether the 11 genetically and morphologically distinct interneuron subtypes also exhibit unique intrinsic physiological properties. Whole-cell patch-clamp recordings were performed for each LTMR-RZ interneuron subtype (Figures 3B and 4B, n = 128 neurons). This analysis revealed that each of the seven types of physiological profiles observed in LTMR-RZ random recordings (Figure 1H) was represented within the genetically labeled interneuron cohorts, with seven profiles associated with excitatory interneuron subtypes (Figure 3B) and five with inhibitory subtypes (Figures 4B and S3F). Moreover, each genetically labeled interneuron subtype exhibited a characteristic firing pattern in response to current injection. For example, within the excitatory cohort, Cbln2^+^ and PKCγ^+^ interneurons are the only populations exhibiting initial bursting and delayed spiking patterns, respectively (Figure 3B). Although reluctant firing profiles were not found in LTMR-RZ random recordings, they represent the most common profile for the excitatory 5HTr6^+^ interneurons (Figure 3B). In contrast to the excitatory cohort, LTMR-RZ inhibitory interneuron subtypes uniquely exhibited either tonic (PVi), delayed (Kcnip2^+^ and Rorβ^+^), or gap firing patterns (Cdh3^+^) (Figure 4B). Thus, the LTMR-RZ comprises seven excitatory and four inhibitory interneuron subtypes, each readily distinguished by unique combination of morphological and physiological properties.

### LTMR-RZ Interneurons Form Axodendritic and Axoaxonic Synapses that Mainly Reside within the LTMR-RZ

**Figure 5:**
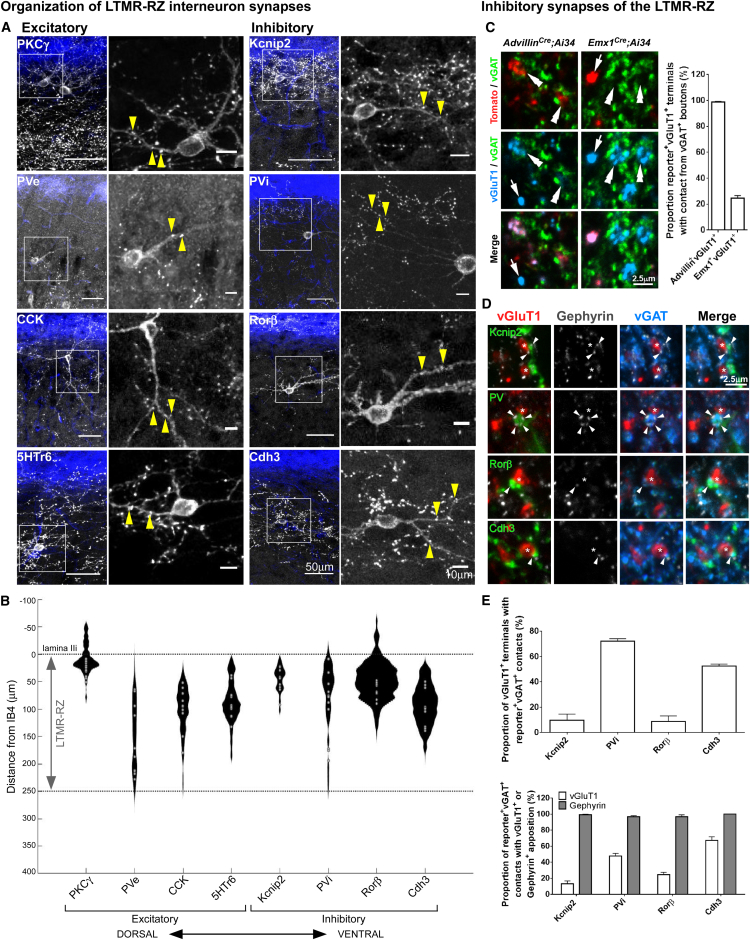
LTMR-RZ Interneurons Make Synapses Largely within the LTMR-RZ and Contribute to Both Pre- and Post-synaptic Inhibition in This Region (A) Images showing synaptophysin-reporter expression driven by recombinase mouse lines to target each interneuron population. IB4 (blue) labels lamina IIi in large-scale view (left panels), with inset magnified in right panels. Arrowheads indicate synaptophysin-reporter^+^ puncta. (B) Violin plots depicting putative synaptic contact number and location by interneuron subtype, as determined by synaptophysin-reporter expression. (C) Images showing synaptophysin-tdTomato (Ai34) expression driven by *Advillin*^*Cre*^ or *Emx1*^*Cre*^ to label sensory or cortical inputs to the LTMR-RZ, respectively. Co-labeling with vGAT and vGluT1 is used to determine axoaxonic contacts onto these terminals, which were quantified across the LTMR-RZ (graph to right). Double arrowheads and arrows indicate vGluT1^+^ terminals with and without vGAT^+^ contacts, respectively. (D) Images showing labeling of PV^+^, Cdh3^+^, Rorβ^+^, and Kcnip2^+^ inhibitory neuron subtype terminals. Co-labeling with vGAT, vGluT1 (asterisks), and gephyrin (arrowheads) is used to determine axoaxonic and axodendritic contacts made by these boutons. (E) Quantification of vGluT1^+^ and gephyrin^+^ apposition to interneuron-reporter^+^vGAT^+^ boutons, representing axoaxonic and axodendritic contacts, respectively. Upper panel displays relative proportion of all vGluT1^+^ boutons in LTMR-RZ receiving axoaxonic contacts from each inhibitory interneuron population. Lower panel displays relative proportion of vGAT^+^ boutons from each inhibitory interneuron population in contact with vGlut1^+^ terminals or gephyrin^+^ puncta. For details of genetic crosses and numbers of cells analyzed, see STAR Methods. See also Figure S5.

**Figure S5:**
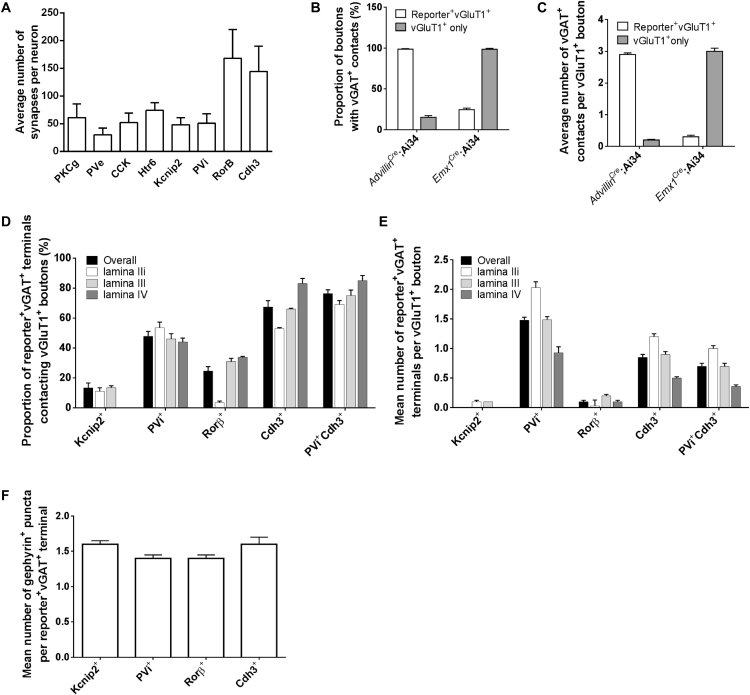
Additional Quantifications of LTMR-RZ Interneuron Synapses, Related to Figure 5 (A) Average number of synapses per neuron for 8/11 LTMR-RZ interneuron populations (n = 3 per population with a minimum of 10 cells per animal). Counts are the same as those used for analysis displayed in Figure 5B. (B) Proportion of Tomato^+^vGluT1^+^ and vGluT1^+^ only terminals receiving vGAT^+^ contacts in *Advillin*^*Cre*^;*R26*^*LSL-synaptophysin-tdTomato*^(Ai34) and *Emx1*^*Cre*^;Ai34 animals (n = 4 for each population). (C) Average number of vGAT^+^ contacts to Tomato^+^vGluT1^+^ and vGluT1^+^ only terminals in *Advillin*^*Cre*^;Ai34 and *Emx1*^*Cre*^;Ai34 animals (n = 4 for each population). (D) Proportion of Reporter^+^vGAT^+^ contacts to vGluT1^+^ boutons as a function of LTMR-RZ lamina (n = 4 for each population). (E) Average number of Reporter^+^vGAT^+^ contacts to individual vGluT1^+^ boutons as a function of LTMR-RZ lamina (n = 4 for each population). (F) Average number of gephyrin^+^ puncta per Reporter^+^vGAT^+^ bouton (n = 3 for each population).

Axodendritic synapses mediate feedforward excitation and inhibition, whereas axoaxonic contacts between inhibitory interneurons and primary afferent terminals provide critical modulation of incoming sensory information through presynaptic inhibition and represent a major component of spinal cord dorsal horn inhibitory circuits (Todd, 1996). Within the LTMR-RZ, we found that inhibitory axoaxonic contacts are largely restricted to vGlutT1^+^ sensory inputs, as descending cortical vGluT1^+^ inputs are associated with few vGAT^+^ appositions (Figures 5C, S5B, and S5C), likely reflecting a lack of axoaxonic contacts (Valtschanoff et al., 1993). To define the type and anatomical localization of excitatory and inhibitory interneuron synapses, we used LTMR-RZ interneuron recombinase tools (Table S1B) in conjunction with recombinase-dependent synaptophysin-reporter mice. We found that the distribution of synapses emanating from individual interneurons (Cdh3^+^, CCK^+^, 5HTr6^+^, PKCγ^+^, Knip2^+^, PVe, PVi, and Rorβ^+^) are predominantly restricted to the LTMR-RZ itself (Figures 5A and 5B), with synapses from individual interneurons largely restricted to lamina in which their cell bodies reside (Figures 5B and S5A). Moreover, we found that sensory neuron vGluT1^+^ axon terminals within the LTMR-RZ receive 2.9 ± 0.1 vGAT^+^ axoaxonic contacts (Figure S5C), and, consistent with previous observations in lamina IIiv and III (Hughes et al., 2012), PVi neurons account for a significant proportion of these (1.5 ± 0.1, Figures 5E and S5E). Cdh3^+^ inhibitory interneurons also contribute many vGAT^+^ axoaxonic contacts within the LTMR-RZ (Figure 5D), while Rorβ^+^ and Kcnip2^+^ inhibitory interneurons form few, if any, axoaxonic contacts in this region (Figures 5E, S5D, and S5E). As previously noted, PVi and Cdh3^+^ interneuron subtypes label an intersecting population (Figure S2D), and PV^+^Cdh3^+^ cells also form axoaxonic contacts in the LTMR-RZ (Figures S5D and S5E). In addition, virtually all Cdh3^+^, Kcnip^+^, Rorβ^+^, and PVi terminals are associated with one or more gephyrin punctum (Figures 5E and S5F), which are most prevalent at axodendritic and axosomatic inhibitory synapses but absent from axoaxonic synapses on primary afferents (Lorenzo et al., 2014). Thus, most and possibly all LTMR-RZ interneuron subtypes synapse locally, within the LTMR-RZ itself, and all of the inhibitory interneuron subtypes make axodendritic synapses, likely to promote feedforward inhibition, while a subset (PVi and Cdh3^+^) form axoaxonic synapses, likely to mediate presynaptic inhibition of primary afferent terminals.

### Each LTMR-RZ Interneuron Subtype Receives Input from LTMRs, Cortex, and Other CNS Sources

**Figure 6:**
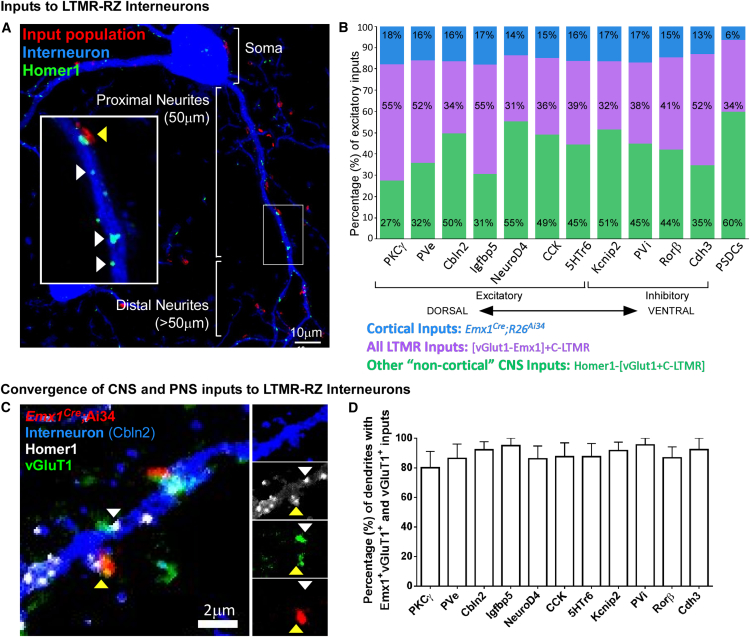
All LTMR-RZ Interneuron Subtypes Receive Inputs from the Periphery, Cortex, and Other CNS Regions (A) Representative image used for anatomical input analysis (Figures 6 and 7). Yellow and white arrowheads indicate excitatory inputs (Homer1^+^ puncta) with and without input from the population of interest, respectively. (B) Compiled quantifications of excitatory inputs from cortex, all LTMRs, and non-cortical CNS onto the 11 interneuron populations and PSDC output neurons. (C) Image showing convergent inputs onto a single dendrite of an interneuron in the LTMR-RZ. Both cortical (Ai34^+^ vGluT1^+^, yellow arrowhead) and sensory (Ai34^−^ vGluT1^+^, white arrowhead) inputs were verified by Homer1^+^ apposition. (D) Relative proportion of dendrites that receive such convergent inputs for all 11 interneuron populations. For further details, see STAR Methods. See also Figure S6.

**Figure S6:**
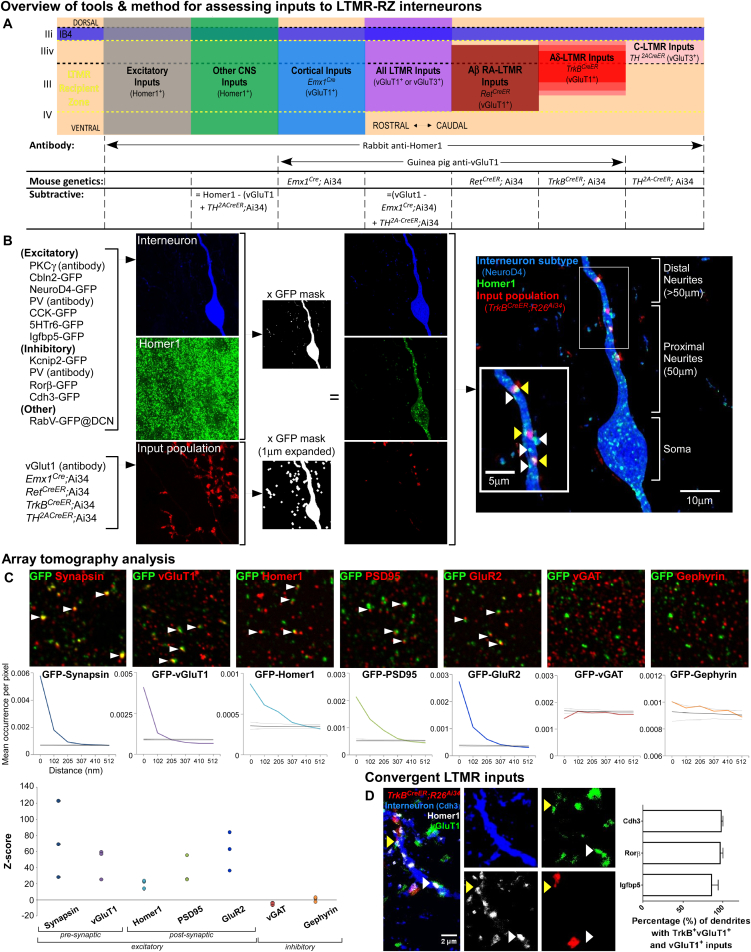
Tools, Approach, and Validation of Anatomically Defined Synapses for Input Analysis, Related to Figures 6 and 7 (A) Overview of genetic tools, antibodies, and subtractive methods used to identify and dissect the relative contributions of various input populations to each interneuron population’s excitatory connectome. Schematic shows relative location of these input populations to the SC DH (sagittal view). Tamoxifen regimens for labeling input populations were as follows: 0.4mg at P21 for *TH*^*2A-CreER*^;*R26*^*LSL-synaptophysin-tdTomato*^(Ai34), 2mg at P21 for *TrkB*^*CreER*^;Ai34, and 2.5mg at E10.5-11.5 for *Ret*^*CreER*^;Ai34. All animals used in this analysis were collected at P30-P40 and lumbar SC was used for analysis. (B) Outline of methods used for quantifying anatomically defined synapses. IHC images were collected and the interneuron channel was used to generate two masks (one containing only interneuron label and the other containing this same region expanded in all directions by 1 μm) that could then be used to isolate only post-synaptic labeling within the interneuron mask and pre-synaptic labeling within the expanded mask. When recombined, counts of inputs with (yellow arrows) and without (white arrows) contacts from the input population of interest were quantified according to cellular compartment (soma, proximal neurite, distal neurite). See STAR Methods. (C) Co-localization analysis of genetically labeled sensory presynaptic axon terminals (*Advillin*^*FlpO*^*;R26*^*FSit*^) using array tomography. Single planes of IHC labeling show association of synaptic markers with GFP^+^ terminals (arrows). Quantifications show mean occurrence of GFP-immunolabeling co-localization per pixel as a function of distance from the center of GFP^+^ boutons. Colored lines represent real data; black and gray lines represent the mean ± standard deviation of randomized data. Z scores for mean marker densities within GFP^+^ terminals for real (n = 3 animals) versus randomized data (n = 4 stacks) indicate higher densities in the real data. (D) IHC image illustrating convergent inputs onto a single dendrite of an interneuron in the LTMR-RZ. Both Aδ-LTMRs (Ai34^+^vGluT1^+^) and other sensory or cortical (Ai34^−^vGluT1^+^) inputs were verified by Homer1^+^ apposition. Quantification shows the relative proportion of dendrites that receive convergent LTMR inputs for three interneuron populations (n = 3 for each interneuron population).

The remarkable degree of LTMR-RZ interneuron subtype diversity raises intriguing questions about allotment of function. Do individual LTMR-RZ interneuron subtypes function as dedicated recipients of particular sensory modalities, or do select LTMR-RZ interneuron subtypes receive inputs from select LTMRs while others receive descending inputs from corticospinal neurons? We visualized LTMR-RZ excitatory synaptic contacts using a combination of genetic tools and synaptic markers (Figure S6A). Putative excitatory synapses were defined as originating from an LTMR population of interest by overlap between pre- and post-synaptic marker proteins (Figures 6A and S6B) and were validated via overlap analysis using array tomography and a range of synaptic markers (Figure S6C). This approach enabled a high-throughput, quantitative analysis of LTMR subtype and cortical neuron synaptic contacts made onto each of the 11 LTMR-RZ interneuron subtypes.

We first compared the amount and distribution of excitatory inputs onto each of the 11 LTMR-RZ populations. Interestingly, each interneuron subtype receives approximately the same density of excitatory synaptic contacts, defined by Homer1^+^ puncta: cell bodies have 0.119 ± 0.003 puncta/μm^2^ (measured as a function of surface area), while proximal and distal dendrites have considerably more synapses, exhibiting 0.836 ± 0.020 and 0.787 ± 0.018 puncta/μm, respectively. We next assessed the number of LTMR, cortical projection neuron, and other “non-cortical” CNS inputs onto each of the 11 genetically labeled LTMR-RZ interneuron subtypes. This analysis revealed that the relative proportions of excitatory inputs onto each of the 11 interneuron subtypes are comparable and range from 30%–55% sensory neuron inputs, 13%–18% cortical projection neuron inputs, and 30%–55% non-cortical CNS inputs (Figure 6B). The non-cortical CNS inputs are likely predominantly vGluT2^+^ synapses from locally projecting interneurons, as Homer1^+^ in the LTMR-RZ is largely accounted for by sensory, cortical, and local interneuron inputs (Figure 1C). Thus, each of the 11 interneuron subtypes receives the majority of its excitatory input from local CNS neurons and/or primary somatosensory neurons, and a lesser, but substantial, number of contacts from corticospinal projection neurons. For all subtypes, a convergence of peripheral nervous system (PNS) and CNS synaptic inputs occurs not just onto interneuron populations as a whole, but onto individual neurons, and often in close proximity on the same dendrite (Figures 6C and 6D). We conclude that each of the 11 LTMR-RZ interneuron subtypes receives convergent inputs originating from LTMRs, cortex, and local spinal cord interneurons.

### LTMR-RZ Interneuron Subtypes Receive Unique Patterns of Convergent LTMR Inputs

**Figure 7:**
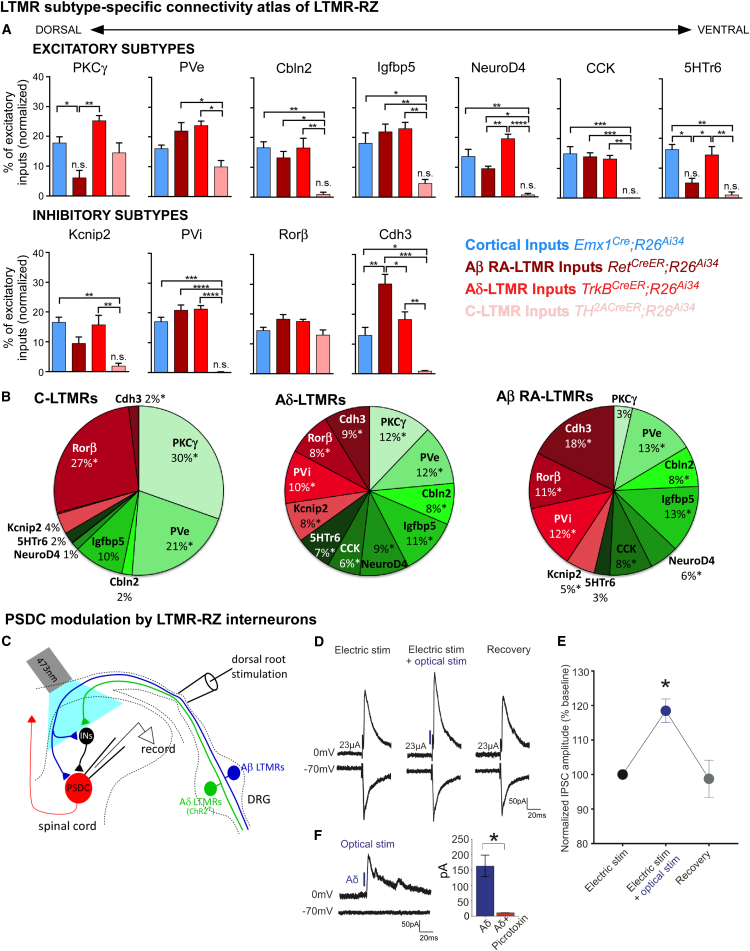
LTMR-RZ Interneuron Subtypes Display Unique Patterns of Tactile Synaptic Inputs (A) Compiled quantifications of excitatory inputs from, left to right, cortex, Aβ RA-LTMRs, Aδ-LTMRs, and C-LTMRs onto each of the 11 interneuron populations (onto proximal+distal neurites). Two-way ANOVA with post hoc Tukey’s test: ^∗^p < 0.05, ^∗∗^p < 0.01, ^∗∗∗^p < 0.0005, ^∗∗∗∗^p < 0.0001. (B) Compiled quantification of LTMR inputs onto the 11 interneuron populations, demonstrating how distinct LTMR subtypes allocate their anatomically defined synapses onto the 11 identified interneuron populations of the LTMR-RZ. ^∗^p < 0.05. (C) Schematic of conditions for PSDC synaptic physiology. (D) Average of 12 consecutive traces showing Aβ-evoked synaptic responses with electrical stimulation of dorsal roots (23 μA) taken just before (left), during (middle), and after (right) optogenetic activation of Aδ-LTMR terminals (blue). (E) Normalized mean inhibitory postsynaptic current (IPSC) amplitude ± SEM; Student’s t test, ^∗^p < 0.05. (F) Left: optical stimulation of Aδ-LTMRs evokes polysynaptic IPSCs onto PSDC neurons. Right: mean optical IPSC in the absence and presence of the GABA_A_R receptor antagonist picrotoxin (100 μM). Student’s t test, ^∗^p < 0.05. For further details on statistical methods, see STAR Methods. See also Figure S6.

While LTMR-RZ interneuron subtypes exhibit comparable proportions of excitatory inputs from sensory neurons, corticospinal neurons, and local CNS neurons (Figure 6B), spinal cord slice electrophysiology experiments using ChR2-assisted circuit mapping demonstrated different levels of synaptic drive from one LTMR subtype, the Aβ RA-LTMR, onto Cbln2^+^, Kcnip2^+^ and Rorβ^+^ interneurons (A.M.C., V.E.A., and D.D.G., unpublished data). Thus, we hypothesized that the number of synaptic contacts derived from select LTMR subtypes is a distinguishing feature of LTMR-RZ interneuron subtypes. To address this possibility, and to generate an LTMR subtype-specific connectivity map of the LTMR-RZ, we quantified putative synaptic contacts between three physiologically distinct LTMR subtypes, C-LTMRs, Aδ-LTMRs, and Aβ RA-LTMRs, and each of the 11 LTMR-RZ interneuron subtypes (Figure S6B and S6C). This analysis revealed that LTMR-RZ interneurons display unique “LTMR synaptic connectivity profiles” (Figure 7A). The relative number of synaptic contacts derived from each of the three individual LTMR subtypes is usually small and comparable to that observed for cortical inputs, on the order of 10%–20% of total excitatory inputs; however, larger variations were also observed, ranging from 0% to 30%. Interestingly, the majority of interneuron subtypes receive input from two or more LTMR subtypes. As observed for cortical neuron and pan-sensory neuron inputs, the convergence of multiple LTMR subtypes onto interneuron subtypes is also evident at the level of individual neurons (Figure S6D). Thus, each of the 11 LTMR-RZ interneuron subtypes samples converging synaptic inputs from at least one, and usually two or more physiologically distinct LTMR subtypes, as well as local interneurons and corticospinal neurons.

The relative proportion of LTMR subtype synapses distributed across each of the 11 interneuron subtypes was also calculated to identify post-synaptic partner preferences for the different LTMRs (Figure 7B). This analysis indicated that C-LTMRs and, to a lesser extent, Aβ RA-LTMRs, exhibit postsynaptic partner selectivity, forming the majority of their synapses onto four of 11 interneuron subtypes and nine of 11 interneuron subtypes, respectively. Importantly, these patterns of synaptic input specificity are not simply a reflection of anatomical organization or the location of LTMR subtype endings and interneuron populations. For example, both Kcnip2^+^ and Cbln2^+^ interneurons reside within the C-LTMR termination zone (Figures 2A and S2B), but neither receives an appreciable number of C-LTMR synaptic contacts. In contrast to the C-LTMR and Aβ RA-LTMR synaptic partner profiles, Aδ-LTMRs divide their synaptic inputs equally across the 11 interneuron subtypes, similar to that of descending cortical inputs. Thus, the majority of LTMR-RZ interneurons receive input from at least two LTMR subtypes, and physiologically distinct LTMR subtypes exhibit a divergence of synaptic contacts onto at least four and as many as 11 LTMR-RZ interneuron subtypes.

### LTMR-RZ Interneurons Modulate Output Pathways that Convey Tactile Information to the Brain

A key to understanding the nature of tactile processing that occurs in the LTMR-RZ is defining the activity of output neurons that carry tactile information to higher brain regions. Thus, we next compared the synaptic connectivity profile of a major LTMR-RZ ascending output population, PSDC neurons, with those of the 11 LTMR-RZ interneuron subtypes. In contrast to each of the 11 LTMR-RZ interneuron subtypes, PSDC neurons receive synaptic inputs largely from local spinal cord interneurons (60%), considerably fewer from sensory neurons (34%), and very few from cortical projection neurons (6%) (Figure 6B). PSDC neurons also receive more restricted types of LTMR synaptic inputs, receiving no contacts from C-LTMRs and fewer synaptic contacts from Aδ-LTMRs than any of the 11 interneuron subtypes (data not shown). Thus, PSDC neurons receive excitatory synaptic inputs mainly from local LTMR-RZ interneurons and, to a lesser extent, Aβ-LTMRs (Figures 6 and 7).

The relatively low number of direct LTMR and cortical inputs and high number of local excitatory inputs onto PSDC neurons suggests a model in which LTMR-RZ interneuron subtypes receive unique combinations of LTMR and cortical inputs and, in turn, connect to PSDC neurons to influence their output activities. Preliminary *ex vivo* recordings of PSDC neurons indicate that these neurons exhibit complex tuning and receptive field properties that are highly distinct from any individual LTMR subtype (A.L.Z. and D.D.G., unpublished data). Thus, we next asked whether PSDC output responses are shaped by combinations of monosynaptic inputs from Aβ-LTMRs and indirect inputs, driven by Aβ-LTMRs, Aδ-LTMRs, and C-LTMRs and conveyed to PSDCs via LTMR-RZ interneurons. Whole-cell patch-clamp recordings of PSDC neurons and electrical stimulation of dorsal roots using a stimulus intensity that selectively activates Aβ fibers revealed the presence of both mono- and polysynaptic inputs onto PSDCs (Figures 7C and 7D). Recordings done with holding potentials at −70 and 0 mV and pharmacological dissection of input properties indicated that the polysynaptic inputs onto PSDCs are both excitatory and inhibitory in nature (Figures 7D and 7F). When the electrical stimulation intensity was increased to activate both Aβ- and Aδ-fibers, we observed an alteration in the polysynaptic waveforms, indicating that inputs from Aδ-fibers are conveyed via LTMR-RZ interneuron polysynaptic connections to PSDCs (data not shown). In complementary experiments, we recorded from PSDC neurons in spinal cord slices expressing ChR2 exclusively in Aδ-LTMRs. Concomitant electrical stimulation of Aβ fibers with optical stimulation of Aδ-LTMR terminals revealed convergent inhibitory polysynaptic inputs from Aβ fibers and Aδ-LTMRs onto PSDC neurons (Figures 7D and 7E). Thus, PSDC neurons receive both direct, monosynaptic Aβ-LTMR synaptic inputs and indirect excitatory and inhibitory inputs via LTMR-RZ interneurons, which are themselves driven by multiple LTMR subtypes and, potentially, cortical projection neurons.

## Discussion

In this study, we report an array of mouse molecular genetic tools that illuminate the cellular and synaptic landscape and organizational logic of the mechanosensory dorsal horn. We found that the LTMR-RZ, which is critical for sensorimotor gating (Bourane et al., 2015) and touch perception (Figures 2D and 2E), exhibits intricate neuronal and synaptic complexity. Our findings point to a highly integrative model of innocuous touch information processing in the spinal cord dorsal horn. In this model, LTMR subtype activity ensembles emanating from the skin, as well as modulatory inputs from the cortex, converge upon 11 LTMR-RZ interneuron subtypes, each serving as a functionally distinct integrator of tactile modalities and descending cortical inputs, to orchestrate patterns of ascending LTMR-RZ projection neuron impulses that underlie touch perception.

### LTMR-RZ Interneuron Diversity and Implications for Innocuous Touch Processing

Defining “cell types” in the nervous system represents a goal for neuroscientists, often aided by a combination of physiological, morphological, biochemical, and functional approaches with the purpose of distinguishing neurons from one another in their contributions to circuits and behavior. Our genetic strategies show that the LTMR-RZ is composed of at least 11 genetically labeled interneuron subtypes that are distinguishable from one another by unique combinations of physiological, morphological, and neurotransmitter properties, as well as patterns of inputs from LTMR subtypes, other LTMR-RZ interneurons, and corticospinal neurons. Cdh3+ inhibitory interneurons, for example, are distinguished by their radial-like morphology, gap action potential discharge patterns, few if any spines, a large number of synaptic inputs from Aβ RA-LTMRs, and they form both axoaxonic and axodendritic synapses. In contrast, PKCγ+ excitatory interneurons exhibit islet-like morphologies, an abundance of spines, delayed firing patterns, and synaptic inputs from Aδ-LTMRs and C-LTMRs, but not an appreciable amount of input from Aβ RA-LTMRs. Our genetic analysis indicates that these 11 subtypes represent 71%–82% coverage of the dorsal horn, though it is likely that additional subtypes remain to be uncovered, in particular, for excitatory cell types. In addition, our classification of subtypes is based on genetic labeling with the purpose of identifying tools that can be implemented to unravel the complexity of this region of the spinal cord in the context of somatosensory circuits and behaviors. As a result of the profound diversity of LTMR-RZ interneuron subtypes, and the circuits they engage, analysis of the LTMR-RZ as a whole, rather than of single interneuron types or lineages, will be needed to advance our understanding of the dorsal horn as a tactile information processing center. It is within this context that the function of LTMR-RZ interneuron subtype morphological, physiological, and synaptic diversity will be revealed.

### LTMR Input Comparisons, Parallel Processing Modules, and Descending Cortical Input Define Innocuous Touch Processing in the Mechanosensory Dorsal Horn

Four principles emerge from our analyses of the cellular and synaptic architecture of the mechanosensory dorsal horn. The first principle is that LTMR-RZ interneuron subtypes receive direct synaptic input from multiple LTMR subtypes in a manner that is not simply a reflection of their laminar positions. PKCγ^+^ interneurons, for example, receive predominantly C-LTMR and Aδ-LTMR input, and negligible Aβ RA-LTMR input, whereas other, neighboring interneuron subtypes receive Aβ RA-LTMR and Aδ-LTMR inputs but few, if any, C-LTMR inputs (Figure 7A). Thus, LTMR-RZ interneuron subtypes sample unique combinations of LTMR inputs, and interneuron outputs may therefore be defined by weighted averages of distinct input modalities. Because LTMR subtypes differ in tuning properties, action potential conduction velocities, receptive field sizes, and adaptation properties, the outputs of LTMR-RZ interneuron subtypes have, in principle, the potential to reflect myriad ensembles of LTMR subtype activities.

A second principle is that of parallel LTMR processing modules, which emerges from two basic observations. First, individual LTMR subtypes diverge to directly contact four or more postsynaptic LTMR-RZ interneuron classes. This is most dramatically exemplified by the synaptic partner profile of Aδ-LTMRs, which show a strikingly even distribution across each of the 11 LTMR-RZ interneuron subtypes described (Figure 7B). Second, in considering the entirety of the excitatory connectome for each LTMR-RZ interneuron type, individual LTMR subclasses represent a minor fraction of the inputs, ranging from 0% to 30% (Figure 7A). This sparse LTMR input allocation distributed broadly across the LTMR-RZ describes a synaptic architecture best exemplified by parallel LTMR input modules. An implication of parallel channels is increased network interconnectivity. In order for a sparse sensory input to perturb a network, there must be sufficient network interconnectivity such that alterations in the activity of a few neurons can spread to other neurons in the network (Laurent, 2002, Rozell et al., 2008). Our observation that the vast majority of synapses formed by LTMR-RZ interneurons reside within the LTMR-RZ itself (Figure 5B), coupled with the finding that the majority of excitatory inputs that form onto all 11 LTMR-RZ interneurons originate in the spinal cord (Figure 1C), indicates a high degree of interconnectivity within the LTMR-RZ. Performing LTMR input computations in parallel rather than hierarchically enables enormous cellular and circuit-level substrate for integration, plasticity, and context-specific output and may enable selective gating of certain modalities under particular physiological states.

The third principle is that excitatory synaptic input from corticospinal neurons is broad and directly engages each LTMR-RZ interneuron (Figure 7A). At the most basic level, the presence of robust cortical input targeting the LTMR-RZ and, remarkably, not the superficial dorsal horn (Figure 1A), suggests that the LTMR-RZ is a locus for somatosensory modulation during conscious tactile exploration. Our observation that cortical inputs are evenly allocated across all interneuron subtypes suggests that cortical activity may have the capacity to influence the gain of all innocuous touch circuit modules. Indeed, electrical activation of somatosensory cortex in cats is sufficient to induce dorsal root potentials, a reflection of presynaptic inhibition (Andersen et al., 1962). Corticospinal projections can thereby engage circuits that modulate gain, presumably through PVi and/or Cdh3^+^ interneurons, which form axoaxonic inhibitory synapses upon LTMR terminals (Figures 5D and 5E). The nature of descending cortical inputs to the LTMR-RZ, which resemble LTMR inputs in terms of both broad distribution of LTMR-RZ interneuron targets and overall numbers of synapses, suggests to us that the LTMR-RZ is a locus for enabling gain modulation during periods of active tactile exploration versus passive touch. We speculate that LTMR-RZ interneurons receive inputs from both LTMRs and cortex to sensitize or desensitize tactile pathways, possibly in a modality-specific and somatotopically organized manner, to differentially process tactile inputs during tactile exploration and passive touch.

The fourth principle to emerge from this study is that LTMR inputs engage LTMR-RZ interneurons and output neurons to the brain in a manner that is essential for touch perception. Inactivation of large cohorts of excitatory and inhibitory LTMR-RZ interneurons revealed that interneurons within this spinal cord region are necessary for perception of texture and normal hairy skin sensitivity (Figures 2D and 2E). PSDC neurons, a major output neuronal population of the LTMR-RZ, receive both direct inputs from Aβ-LTMRs and indirect inputs from LTMR-RZ interneurons, which are themselves synaptic partners of two or more LTMR subtypes as well as cortical neurons. Electrophysiological recordings of PSDC neurons revealed them to be activated directly by Aβ-LTMRs and indirectly by Aβ-LTMRs, Aδ-LTMRs, and possibly C-LTMRs, via excitatory and inhibitory LTMR-RZ interneurons (Figures 7D and 7F). These findings, taken together, indicate that processing of innocuous touch information relevant for perception begins in the LTMR-RZ and is conveyed to the brain via postsynaptic ascending pathways. Thus, we propose an integrative model for touch information processing in which LTMR activity ensembles emanating from the skin and descending modulatory inputs from the cortex converge upon an array of LTMR-RZ excitatory and inhibitory networks. These networks are composed of 11 or more morphologically, physiologically, and synaptically distinct LTMR-RZ interneuron subtypes that function to sculpt the activity of ascending pathways, which, together with the direct dorsal column pathway, underlie tactile discrimination and perception.

## STAR★Methods

### Key Resources Table

**Table:** 

REAGENT or RESOURCE	SOURCE	IDENTIFIER
**Antibodies**		

647-IB4 (1:500, IHC)	Invitrogen	I32450
Rabbti anti-CCK (1:1000, IHC)	Frontier Institute	CCK-pro-Rb-Af350
Mouse anti-gephyrin(7A) (1:500, IHC)	SynapticSystems	147 021
Rabbit anti-gephyrin (1:100, AT)	BD Biosciences	612632; RRID: AB_399669
Chicken anti-GFP (1:100, AT)	GeneTex	GTX13970; RRID: AB_371416
Chicken anti-GFP (1:1000, IHC, WM)	Aves	GFP 1020; RRID: AB_10000240
Chicken anti-GFP (1:1000, IHC)	Abcam	13970
Rabbit anti-GFP (1:1000, IHC, WM)	Invitrogen	A11122; RRID: AB_221569
Mouse anti-GluR2 (1:50, AT)	Millipore	MAB397; RRID: AB_2113875
Rabbit anti-dsRed (1:1000, IHC, WM)	Clontech	632496; RRID: AB_10013483
Rabbit anti-Homer1 (1:500, AT; 1:1000, IHC)	Synaptic Systems	160 003
Goat anti-mCherry (1:500, WM)	Sicgen	Ab0040-200; RRID: AB_2333092
Chicken anti-NFH (1:1000, IHC)	Aves	NFH0211
Rabbit anti-NF200 (1:1000, WM)	Sigma	N4142; RRID: AB_477272
Mouse anti-NeuN (1:1000, IHC	Millipore	MAB377; RRID: AB_2298772
Goat anti-Parvalbumin (PV) (1:2000, IHC)	SWANT	PVG-213
Guinea Pig anti-Parvalbumin (PV) (1:500, IHC)	Frontier Institute	PV-GP-Af1000; RRID: AB_2336938
Rabbit anti-Parvalbumin (PV) (1:2000-1:3000, IHC)	SWANT	PV-25; RRID: AB_10000344
Rabbit anti-PKCg (1:1000, IHC)	Santa Cruz	sc-211; RRID: AB_632234
Guinea Pig anti-PKCg (1:1000, IHC)	Frontier Institute	PKCg-GP-Af350
Goat anti-PKCg (1:500, IHC)	Frontier Institute	PKCg-Go-Af840
Mouse anti-PSD95 (1:100, AT)	NeuroMab	75-028; RRID: AB_2307331
Rabbit anti-Synapsin1 (1:100, AT)	Millipore	AB1543; RRID:AB_2200400
Rat anti-Troma1 (1:50, WM)	DSHB (U of Iowa)	TROMA-I; RRID: AB_531826
Goat anti-vGAT (1:1000, IHC)	Frontier Institute	VGAT-Go-Af620
Mouse anti-vGAT (1:100, AT; 1:1000, IHC)	Synaptic Systems	131 011; RRID: AB_887868
Guinea Pig anti-vGluT1 (1:1000-1:5000, IHC, AT)	Millipore	AB5905; RRID: AB_2301751
Rabbit anti-vGluT1 (1:20000, IHC)	Synaptic Systems	135 303; RRID: AB_887874

**Experimental Models: Organisms/Strains**		

Mouse: Cbln2-GFP (see Table S1A for genotyping primers and additional information)	GENSAT	BAC address: RP23-168P8-GFP
Mouse: Cdh3-GFP (see Table S1A for genotyping primers and additional information)	GENSAT	BAC address: RP23-199J15-GFP
Mouse: Cdh3-CreER (see Table S1B for genotyping primers and additional information)	This paper	BAC address: RP23-267K22-CreERT2
Mouse: CCK-GFP (see Table S1A for genotyping primers and additional information)	GENSAT	BAC address: RP23-234I17-GFP
Mouse: CCK^CreER^ (see Table S1B for genotyping primers and additional information)	Jackson Laboratories	JAX#012710
Mouse: 5HTr6-GFP (see Table S1A for genotyping primers and additional information)	GENSAT	BAC address: RP23-65B16-GFP
Mouse: 5HTr6-CreER (see Table S1B for genotyping primers and additional information)	This paper	BAC address: RP23-65B16-CreERT2
Mouse: Igfbp5-GFP (see Table S1A for genotyping primers and additional information)	GENSAT	BAC address: RP24-159O10-GFP
Mouse: Kcnip2-GFP (see Table S1A for genotyping primers and additional information)	GENSAT	BAC address: RP23-146N4-GFP
Mouse: Kcnip2-CreER (see Table S1B for genotyping primers and additional information)	This paper	BAC address: RP23-146N4-CreERT2
Mouse: NeuroD4-GFP (see Table S1A for genotyping primers and additional information)	GENSAT	BAC address: RP23-55O18-GFP
Mouse: *PKCγ*^*GFP*^	This paper	MGI: 97597
Mouse: *PKCγ*^*CreER*^	This paper	MGI: 97597
Mouse: PV-tdTomato	Kaiser et al., 2016	MGI: 97821
Mouse: *PV*^*FlpO*^	Jackson Laboratories	JAX#022730
Mouse: *PV*^*2A-CreER*^	Jackson Laboratories	JAX#028580
Mouse: *Rorβ*^*GFP*^ (see Table S1A for genotyping primers and additional information)	Liu et al., 2013	MGI: 5548299
Mouse: *Rorβ*^*CreER*^ (see Table S1B for genotyping primers and additional information)	This paper	MGI: 5548299
Mouse: *R26*^*CreER*^	Jackson Laboratories	JAX#004847
Mouse: *R26*^*LSL-YFP*^ (Ai3)	Jackson Laboratories	JAX#007903
Mouse: *R26*^*LSL-tdTomato*^ (Ai9)	Jackson Laboratories	JAX#007909
Mouse: *R26*^*LSL-ChR2-YFP*^ (Ai32)	Jackson Laboratories	JAX#007909
Mouse: *R26*^*synaptophysin-tdTomato*^ (Ai34)	Jackson Laboratories	JAX#012570
Mouse: *R26*^*LSL-FSF-tdTomato*^ (Ai65)	Jackson Laboratories	JAX#021875
Mouse: *RC::FPsit*	Niederkofler et al., 2016	See STAR Methods
Mouse: *RC:FPtox*	Kim et al., 2009	See STAR Methods
Mouse: *Advillin*^*Cre*^	Hasegawa et al., 2007	MGI:1333798
Mouse: *Emx1*^*Cre*^	Jackson Laboratories	JAX#005628
Mouse: *Lbx1*^*Cre*^	Sieber et al., 2007	MGI: 104867
Mouse: *vGAT*^*iresCre*^	Jackson Laboratories	JAX#016962
Mouse: *GAD2*^*2A-mCherry*^	Jackson Laboratories	JAX#023140
Mouse: vGluT2-YFP	Jackson Laboratories	JAX#017978
Mouse: *GAD67*^*GFP*^	Tamamaki et al., 2003	MGI: 95632
Mouse: GlyT2-GFP	Punnakkal et al., 2014	MGI: 105090
Mouse: *Ret*^*CreER*^	Luo et al., 2009	MGI: 97902
Mouse: *Ret*^*fCFP*^	Uesaka et al., 2008	MGI: 97902
Mouse: *TrkB*^*CreER*^	Rutlin et al., 2015	MGI: 97384
Mouse: *TrkC*^*CreER*^	Bai et al., 2015	MGI: 97385
Mouse: *TH*^*2A-CreER*^	This paper	MGI: 98735

**Software and Algorithms**		

ImageJ Puncta Analyzer Plugin	Ippolito and Eroglu, 2010	imagej.nih.gov/ij
Spot and puncta detection & co-localization analysis for array tomography (MATLAB scripts)	Saunders et al., 2015	available upon request (lab of Dr. Bernardo Sabatini)
Neurolucida 360	MBF Biosciences	http://www.mbfbioscience.com/neurolucida360

### Contact for Reagent and Resource Sharing

Please contact the Lead Contact David Ginty at Harvard Medical School, david_ginty@hms.harvard.edu, with any request regarding reagents used in this study.

### Experimental Model and Subject Details

#### Animals

Mouse lines generated and analyzed for dorsal horn interneuron expression are described in Table S1B. Other published mouse lines used include *CCK*^*iresCre*^ (Jax#012706); *Rorβ*^*iresCre*^ (Jax#023526); *PV*^*2A-CreER*^ (Jax#028580); *R26*^*CreER*^ (Jax#004847); *R26*^*LSL-YFP*^(Ai3) (Jax#007903); *R26*^*LSL-tdTomato*^(Ai9) (Jax#007909); *R26*^*LSL-FSF-TdTom*^ (Ai65) (Jax#021875); *R26*^*LSL-synaptophysin-tdTomato*^ (Ai34) (Jax#012570); *R26*^*LSL-ChR2-YFP*^(Ai32) (Jax#012569); *Advillin*^*Cre*^ (Hasegawa et al., 2007); *Emx1*^*Cre*^ (Jax#005628 (Gorski et al., 2002)); *Lbx1*^*Cre*^ (Sieber et al., 2007); *vGlut2*^*iresCre*^ (JAX#016963); *vGAT*^*iresCre*^ (JAX#016962); *GAD2*^*2A-mCherry*^ (JAX#023140); vGluT2-YFP (JAX#017978); *GAD67*^*GFP*^ (Tamamaki et al., 2003); GlyT2-GFP (Punnakkal et al., 2014); *RC::FPSit* (Niederkofler et al., 2016); *RC::PFtox* (Kim et al., 2009). Published LTMR-CreER lines include *TrkB*^*CreER*^ (Rutlin et al., 2015); *Ret*^*CreER*^ (Luo et al., 2009); *TrkC*^*CreER*^ (Bai et al., 2015), and *Ret*^*fCFP*^ (Uesaka et al., 2008). Mice were handled and housed in accordance with the Harvard Medical School and Johns Hopkins University IACUC guidelines. For histological experiments P30-35 male and female mice were used. For electrophysiological experiments P13-P21 male and female mice were used. For behavioral experiments 7 week old male mice were used.

The following BAC transgenics and targeted alleles were generated for this study. The Cdh3-CreER (NIDA158), 5HTr6-CreER (NIDA108) and Kcnip2-CreER (NIDA099) BAC transgenic mouse lines were generated by introducing a 4.7 kb CreERT2 cassette, pLD53.CreERT2, into the following bacterial artificial chromosomes, RP23-267K22 (Cdh3); RP23-65B16 (5HTr6) and RP23-146N4 (Kcnip2). A detailed step-by-step description of the BAC modification method has been published previously (Gong et al., 2010). Briefly, CreERT2 was inserted at the start site of the gene of interest via a two plasmid/ one-recombination step process. The modified BACs were expanded in *E. coli*, linearized by PI-SceI and microinjected in the pronuclei of fertilized C57BL/6 J embryos. In the case of RP23-146N4 (Kcnip2) linearization was done with NotI, instead of PI-SceI and the DNA subsequently run through a CL-4B hydrophobic interaction column. The *PKCγ*^*myrGFP*^ and *PKCγ*^*CreERT2*^ knock-in mice were generated using a targeting vector that was made utilizing a 2-step recombineering protocol (Liu et al., 2003). The genomic sequence of mouse PKCγ gene (strain 129S7/SvEv) was obtained from Ensembl Mouse Genome Browser. A 184kb BAC clone (bMQ_233p05) containing exon 1 of the *PKCγ* gene was obtained from SourceBioScience. A 4.7kb region (2.2kb-pre and 2.5kb-post first coding ATG of exon 1) from bMQ_233p05 was subcloned into a pBluescript-diptheria toxin A (PBS-DTA) plasmid via a first recombineering step. A myristoylated GFP (myrGFP) and Cre recombinase- estrogen receptor T2 (CreERT2) fusion -*Frt-Neomycin-Frt-loxP* cassette was introduced into the first coding ATG of exon 1 of the *PKCγ* gene via a second recombineering step. The *Rorβ*^*CreERT2*^ knock-in mice were generated using a targeting vector that was made utilizing a 2-step recombineering protocol (Liu et al., 2003). The genomic sequence of mouse *Rorβ* (strain NOD/LtJ) was obtained from Ensembl Mouse Genome Browser. A 167kb BAC clone (CH29-604B6) containing exon 1 of the *Rorβ* gene was obtained from CHORI. A 9.7kb region (1.3kb-pre and 8.4kb-post first coding ATG of exon 1) from CH29-604B6 was subcloned into a pBluescript-diptheria toxin A (PBS-DTA) plasmid via a first recombineering step. A *Cre recombinase-estrogen receptor T2* (CreERT2) fusion-*Frt-Neomycin-Frt-loxP* cassette was introduced into the first coding ATG of exon 1 of the *RORβ* gene via a second recombineering step. The last 4bp of *RORβ* exon 1 were replaced. *TH*^*2A-CreER*^ knock-in mice were generated using a targeting vector that was made utilizing a 2-step recombineering protocol (Liu et al., 2003). The genomic sequence of mouse *TH* (strain 129S7/SvEv) was obtained from Ensembl Mouse Genome Browser. BAC clone bMQ_453O04 containing exon 13 of the *TH* gene was obtained from SourceBioScience. A 9.15kb region (5kb pre-3′UTR and 4.1kb including and post 3′UTR) from bMQ_453O04 was subcloned into a pBluescript-diptheria toxin A (PBS-DTA) plasmid via a first recombineering step. A Cre recombinase- estrogen receptor T2 (CreERT2) cassette was introduced after a T2A peptide coding sequence immediately before the start of the 3′UTR of the tyrosine hydroxylase gene, and a *Frt-Neomycin-Frt-loxP* introduced directly after the 3′UTR via a second recombineering step. The *Advillin*^*FlpO*^ knock-in mice used in experiments shown in Figure 1D will be described elsewhere (T. Dickendesher and D.D.G., unpublished data). Cdx2-NSE-FlpO BAC transgenic animals were generated from the previously described vector for generation of the Cdx2-NSE-Cre BAC transgenic line (Coutaud and Pilon, 2013) by replacing the Cre cassette with FlpO using standard cloning techniques. Cdx2-NSE-FlpO transgenic DNA was microinjected in FVB/N oocytes in accordance to standard methods. Offspring were screened for PCR-based genotyping of tail DNA using specific FlpO recombinase primers (forward within NSE sequence: 5′TAGCCAGACTCCTGCCTGAT3′, reverse within FlpO sequence: 5′GTTCACGATGTCGAA GCTCA3′). Eight F0 transgenic animals were identified, of which only males (four in total) were tested for FlpO activity. FlpO activity was evaluated by crossing F1 male mice with *R26*^*FSF-GFP*^ female animals and setting timed pregnancies using standard vaginal plug detection with noon of the day of plug considered at E0.5. Embryos at embryonic day 12.5 were collected and screened for caudal expression (as described in Figure S2A). Of the four F0 lineages tested, one resulted in the correct recombination pattern. Two F1 males from this lineage were kept to propagate the Cdx2-NSE-FlpO mouse line via breeding with FVB/N females. Of note, Cdx2-NSE-FlpO transgene expression is sensitive to background, as described in (Coutaud and Pilon, 2013), this likely reflects the fact that regulatory sequences used to generate this transgene were cloned from FVB genomic DNA.

### Method Details

#### Genetic Crosses and statistical methods related to individual figures

Genetic crosses related to Figure 1: (A) C-LTMR inputs labeled with *TH*^*2A-CreER*^*;R26*^*LSL-synaptophysin-tdTom*^(Ai34) (0.5 mg tamoxifen treatment at P21); Aδ-LTMRs inputs labeled with *TrkB*^*CreER*^*;*Ai34 (2 mg tamoxifen at P21); Aβ RA-LTMRs labeled with *Ret*^*CreER*^*;*Ai34 (2 mg tamoxifen at E10.5-11.5); Aβ SAI-LTMRs and Aβ Field-LTMRs labeled with a *TrkC*^*CreER*^;*Ret*^*fCFP*^ intersectional strategy (3 mg tamoxifen at E13.5 to label Aβ SAI-LTMRs and 2 mg tamoxifen at P21 to label Aβ Field-LTMRs). Cortical inputs are labeled with *Emx1*^*Cre*^*;*Ai34. (B) PSDCs were labeled in a retrograde fashion as described below with CTB555. (C) Spinal cord inputs are labeled with *Lbx1*^*Cre*^, sensory inputs with *Advillin*^*Cre*^, and cortical inputs with *Emx1*^*Cre*^. All lines are crossed to Ai34 to visualize inputs. *Lbx1*^*Cre*^ accounts for 94.75 ± 0.96% of all NeuN^+^ cells in the LTMR-RZ, indicating that these inputs are largely emanating from locally-projecting spinal cord interneurons. (n = 3 for each input population and animals counted). (D) Sensory inputs are labeled with *Advillin*^*Cre*^*;*Ai34. Cortical inputs are labeled with *Emx1*^*Cre*^*;*Ai34. Proprioceptive inputs are labeled with *PV*^*2A-CreER*^*;Advillin*^*FlpO*^*;R26*^*FPSit*^ (2 mg tamoxifen at P21). (F) Excitatory neurons are labeled with *vGluT2*^*ires-Cre*^*;R26*^*LSL-YFP*^(Ai3) or *vGluT2*^*YFP*^. Inhibitory neurons are labeled with *vGAT*^*ires-Cre*^*;*Ai3, *GAD65*^*mCherry*^, and GlyT2-GFP. PSDC neurons are labeled retrogradely from the dorsal columns and lateral parabrachial nucleus with CTB555, respectively. Quantification depicted as percentage of total NeuN^+^ neurons within the LTMR-RZ. (G) Interneurons labeled randomly with *R26*^*CreER*^*;*Ai3 and 100 μg of tamoxifen at E13.5.

Genetic crosses and statistical methods related to Figure 2: (A) The following number of cells and animals were used for this analysis; Cbln2: 633 GFP^+^ neurons counted (n = 3 animals); Cdh3: 201 GFP^+^ neurons counted (n = 3 animals); CCK: 243 GFP^+^ neurons counted (n = 6 animals); 5HTr6: 350 GFP^+^ neurons counted (n = 3 animals); Igfbp5: 592 GFP^+^ neurons counted (n = 3 animals); Kcnip2: 487 GFP^+^ neurons counted (n = 3 animals); NeuroD4: 155 GFP^+^ neurons counted (n = 4 animals); PKCγ: 471 PKCγ^+^ neurons counted (n = 3 animals); PV: 320 TdTom^+^ neurons counted (n = 3 animals); Rorβ: 437 GFP^+^ neurons counted (n = 3 animals). (B) For each cross at least three animals were analyzed with at least 100 GFP^+^ cells counted per animal. For Cdh3 (*vGlut2*^*iresCre*^ or *vGAT*^*iresCre*^*; R26*^*LSL-TdTom*^(Ai9)*;*Cdh3-GFP); Cbln2 (*vGlut2*^*iresCre*^ or *vGAT*^*iresCre*^*; R26*^*LSL-TdTom*^(Ai9)*;*Cbln2-GFP); CCK (*vGlut2*^*iresCre*^ or *vGAT*^*iresCre*^*; R26*^*LSL-TdTom*^(Ai9)*;*CCK-GFP); 5HTr6 (*vGlut2*^*iresCre*^ or *vGAT*^*iresCre*^*; R26*^*LSL-TdTom*^(Ai9)*;*5HTr6-GFP); Igfbp5 (*vGlut2*^*iresCre*^ or *vGAT*^*iresCre*^*; R26*^*LSL-TdTom*^(Ai9)*;*Igfbp5-GFP); Kcnip2 (*vGlut2*^*iresCre*^ or *vGAT*^*iresCre*^*; R26*^*LSL-TdTom*^(Ai9)*;*Knip2-GFP); NeuroD4 (*vGlut2*^*iresCre*^ or *vGAT*^*iresCre*^*; R26*^*LSL-TdTom*^(Ai9)*;*NeuroD4-GFP); PKCγ (*vGlut2*^*iresCre*^ or *vGAT*^*iresCre*^*; R26*^*LSL-TdTom*^(Ai9) with Rb anti-PKCγ antibody, see antibody list Table S1); PV (*vGlut2*^*iresCre*^ or *vGAT*^*iresCre*^*; R26*^*LSL-TdTom*^(Ai9) with Rb anti-PV antibody, see antibody list Table S1); Rorβ (*vGlut2*^*iresCre*^ or *vGAT*^*iresCre*^*; R26*^*LSL-TdTom*^(Ai9)*;Rorβ*^GFP^). (E) For *CCK*^*iresCre*^*;*Cdx2-NSE-FlpO*;RC::PFTox* animals (top) 100ms ISI tactile PPI results displayed (main effect of genotype across all ISIs, two-way ANOVA: ^∗^p < 0.05, F[1,65] = 8.578). For *Rorβ*^*iresCre*^*;*Cdx2-NSE-FlpO*;RC::PFTox* (bottom) 50ms ISI tactile PPI displayed (main effect of genotype across all ISIs, two-way ANOVA: ^∗^p < 0.05, F[1,125] = 5.717).

Genetic crosses related to Figure 3: (A and A′) The following mouse crosses were used to label interneuron populations for Neurolucida reconstructions, at least 3 animals per cross were used for analysis: PKCγ (*PKCγ*^*CreER*^*;R26*^*LSL-YFP*^(Ai3) 2mg of tamoxifen at P15); Cbln2 (Cbln2-GFP); NeuroD4 (NeuroD4-GFP); PVe (*PV*^*FlpO*^*;vGluT2*^*iresCre*^*;R26*^*LSL-FSF-TdTom*^(Ai65)); CCK (*CCK*^*CreER*^*;R26*^*LSL-YFP*^(Ai3)) 2mg of tamoxifen at P21); 5HTr6 (5HTr6-GFP); Igfbp5 (Igfbp5-GFP). Number of cells reconstructed: PKCγ (n = 31); Cbln2 (n = 25); NeuroD4 (n = 17); PVe (n = 28); CCK (n = 33); 5HTr6 (n = 29); Igfbp5 (n = 41). (B and B′) The following mouse crosses were used to label each interneuron population for electrophysiology, at least 3 animals per cross were used for analysis: PKCγ (*PKCγ*^*CreER*^*;R26*^*LSL-YFP(Ai3)*^ 2mg of tamoxifen at P15); Cbln2 (Cbln2-GFP); NeuroD4 (NeuroD4-GFP); PVe (*PV*^*FlpO*^*;vGluT2*^*iresCre*^*;R26*^*LSL-FSF-TdTom*^(Ai65)); CCK (*CCK*^*CreER*^*;R26*^*LSL-YFP*^(Ai3) 2mg of tamoxifen at P15); 5HTr6 (5HTr6-GFP); Igfbp5 (Igfbp5-GFP). Total number of neurons recorded from each cross: PKCg (n = 7); Cbln2 (n = 27); NeuroD4 (n = 10); PVe (n = 10); CCK (n = 10); 5HTr6 (n = 10); Igfbp5 (n = 9). RF = Reluctant Firer, SS = single spiking, IB = Initial Bursting, p = Phasic, G = Gap, D = Delayed, RS = Regular Spiking; T = Tonic.

Genetic crosses related to Figure 4: (A)The following mouse crosses were used to label interneuron populations for Neurolucida reconstructions, at least 3 animals per cross were used for analysis: PVi (*PV*^*FlpO*^*;vGAT*^*iresCre*^*; R26*^*LSL-FSF-TdTom*^(Ai65)); Kcnip2 (Kcnip2-GFP); Rorβ (*Rorβ*^*CreER*^;*R26*^*LSL-YFP*^(Ai3) 2mg of tamoxifen at P18); Cdh3 (Cdh3-GFP). Number of cells reconstructed: PVi (n = 31); Kcnip2 (n = 41); Rorβ (n = 43); Cdh3 (n = 32). (B) The following mouse crosses were used to label each interneuron population for electrophysiology, at least 3 animals per cross were used for analysis: PVi (*PV*^*FlpO*^*;vGAT*^*iresCre*^*; R26*^*LSL-FSF-TdTom*^(Ai65)); Kcnip2 (Kcnip2-GFP); Rorβ (*Rorβ*^*GFP*^); Cdh3 (Cdh3-GFP). Total number of neurons recorded from each cross: PVi (n = 9); Kcnip2 (n = 12); Rorβ (n = 12); Cdh3 (n = 12). RF = Reluctant Firer, SS = single spiking, IB = Initial Bursting, p = Phasic, G = Gap, D = Delayed, RS = Regular Spiking; T = Tonic.

Genetic crosses related to Figure 5: (A) The following mouse crosses were used to label each interneuron population for input analysis: PKCγ^+^ (*PKCγ*^*CreER*^*;*Ai34), PVe(*PV*^*FlpO*^*;vGluT2*^*Cre*^*;R26*^*FPSit*^), CCK^+^ (*CCK*^*CreER*^*;*Ai34), 5HTr6^+^ (5HTr6-CreER*;*Ai34), Kcnip2^+^ (Kcnip2-CreER*;*Ai34), PVi^+^ (*PV*^*FlpO*^*;vGAT*^*Cre*^*;R26*^*FPSit*^), Rorβ^+^ (*Rorβ*^*CreER*^*;*Ai34), and Cdh3^+^ (Cdh3-CreER*;*Ai34) interneuron subtypes. Mice were treated with 0.5-2mg tamoxifen at P21. (B) At least 3 animals (10 neurons total) were used for this analysis for each interneuron subtype, using the same crosses described above. Black plots indicate presence of synapses at specific dorsal-ventral locations (distance from IB4, y axis) and the relative number of synapses at those levels (depicted by plot width) for the 10 cells of each interneuron type (soma location plotted with gray circles). (C) At least 4 animals of each reporter line was used for this analysis. (D) The following mouse crosses were used for this analysis: Cdh3^+^ (Cdh3-GFP), Rorβ^+^ (*Rorβ*^*CreER*^*;*Ai34), Kcnip2^+^ (Kcnip2-CreER*;*Ai34), and PV^+^ (immunostaining).

Experimental details related to Figure 6: (A) Image prepared as outlined in STAR Methods and Figure S6B. Image shown here is maximum intensity projection across a depth of 5 μm, although analysis was never conducted on projections. (B) Quantitative data presented are for proximal and distal neurites only, as somatic inputs were minimal in all subtypes and no significant difference as a function of proximal versus distal was observed in overall excitatory input (as measured by Homer1^+^) or broad input quantifications. (n = 3 for each interneuron and input population combination).

Statistical methods for Figure 7: (A) Values are normalized percentages of excitatory input as measured by Homer1^+^ puncta (n = 3 for each interneuron and input population combination. See STAR Methods). To determine if inputs are truly above 0, a one-sample t test was used. If input values were not statistically significantly (p > 0.05) above 0%, lack of significance was indicated by “n.s.” above the respective bar graph. For comparisons between input lines onto individual interneuron populations, statistics are denoted above bars by brackets. For PKCγ: (one-way ANOVA: p = 0.0042, F[3,8] = 10.19). For Cbln2: (one-way ANOVA: p = 0.0031, F[3,8] = 11.16). For NeuroD4: (one-way ANOVA: p = 0.0001, F[3,8] = 27.46). For PVe: (one-way ANOVA: p = 0.0110, F[3,7] = 8.143). For CCK: (one-way ANOVA: p = 0.0003, F[3,8] = 21.63). For 5HTr6: (one-way ANOVA: p = 0.0012, F[3,8] = 15.08). For Igfbp5: (one-way ANOVA: p = 0.0031, F[3,8] = 11.23). For Kcnip2: (one-way ANOVA: p = 0.0046, F[3,8] = 9.874). For PVi: (one-way ANOVA: p < 0.001, F[3,7] = 55.47). For Rorβ: (one-way ANOVA: p = 0.0.678, F[3,8] = 3.540). For Cdh3: (one-way ANOVA: p = 0.0002, F[3,8] = 24.45). (B) Contributions from LTMR input populations that were found to be greater than 0% of an interneuron population’s excitatory input (using a one-sample t test) are denoted with asterisks. (n = 3 for each interneuron and input population combination). (D) At *V*_*h*_ = 0mV, optical stimulation of Aδ-LTMRs increases the amplitude of the feedforward polysynaptic Aβ-evoked IPSCs (n = 4/4 cells). At *V*_*h*_ = −70mV, the electrical evoked EPSC does not change with concomitant optical stimulation (5ms pulses during electrical stimulation; blue rectangle; n = 9 PSDC neurons).

#### Tamoxifen treatment

Tamoxifen was dissolved in ethanol (20 mg/ml), mixed with an equal volume of sunflower seed oil (Sigma), vortexed for 5-10 min and centrifuged under vacuum for 20-30 min to remove the ethanol. The solution was kept at −20°C and delivered via oral gavage to pregnant females for embryonic treatment (E10-5-E13.5, as specified in the figure legends) or via intraperitoneal injection or oral gavage for postnatal treatments (P8-P25, as specified in the figure legends). For all analyses, the morning after coitus was designated as E0.5 and the day of birth as P0.

#### Dorsal column injections for PSDC labeling

Male and female mice (P13-15) were anesthetized via continuous inhalation of isoflurane (1%–3%) from a precision vaporizer for the 30-60 min duration of the surgery. The animal’s breathing rate was monitored throughout the procedure and the anesthetic dose was adjusted as necessary. Puralube eye ointment was applied to the eyes. The back of the neck was shaved, treated with commercial depilatory cream (NAIR, Church and Dwight Co.; Princeton, NJ) for 0.5-1 min, and swabbed with water and Betadine. A 5 mm incision was made in the midline of the back skin at the cervical level and local anesthetic (0.5% lidocaine) was applied to the incision site. Muscles were cut or separated from the midline until the spinal cord cervical vertebrae were exposed. A small incision was made on the dura and arachnoid membranes immediately above the C1 cervical spinal vertebrae to expose the DCN. 100-200 nL of Adeno-Associated Virus (AAV2/1-CMV-Cre, titer 9.78e12 in 0.9% saline, Penn Vector Core), Rabies Virus (RabV-deltaG-GFP, titer 5.84E+7 - 9.48E+8 IU/mL, Salk Institute or Boston Children’s viral core), or 100-300nl of CTB555 (2 μg/μl in PBS, Invitrogen) was injected into the DCN using a glass pipette under visual guidance. Afterward, muscles and skin were stitched together with sutures, and Carprofen (4 mg/kg) was injected subcutaneously for analgesia. Mice recovered from anesthesia on a warm pad for 1 hr and were returned to their home cage (housed in groups of 5). Additional doses of Carprofen were injected intraperitoneally at 24 and 48 hr post-operation. The condition of the mice, including the healing of wounds, body weight, and grooming, was monitored daily. At the appropriate time point (4 weeks following AAV injections or 3-7 days following CTB or RabV injections), mice were sacrificed by CO_2_ asphyxiation followed by perfusion, or used for electrophysiology experiments.

#### Spinal cord slice preparation and electrophysiological recordings

Acute spinal cord sagittal slices were used for whole-cell patch clamp recordings of dorsal horn interneurons. Male and female mice (P14-P21) were briefly anesthetized via continuous inhalation of isoflurane (1%–3%) while the spinal column was removed. On cold choline solution (92mM Choline Chloride, 2.5mM KCL, 1.2mM NaH_2_PO_4_, 30mM NaHCO_3_, 20mM HEPES, 2.5mM Glucose, 5mM Sodium Ascorbate, 2mM Thiourea, 3mM Sodium Pyruvate, 10mM MgSO_4_ 7H_2_O, 0.5mM CaCl_2_ 2H_2_O) the lumbar enlargement was removed from the spinal column and mounted in 0.3% LMP agar for slicing in the sagittal plane (250-400um, Leica VT1200S). Spinal cord slices were allowed to recover at 34°C for 30 min in ACSF containing 2.5mM CaCl2, 1mM NaH2PO4, 119mM NaCl, 2.5mM KCl, 1.3mM MgSO_4_ 7H_2_O, 26mM NaHCO_3_, 25mM dextrose, and 1.3mM Na ascorbate, saturated with 95% O_2_, 5% CO_2_ at a rate of ∼2 ml/min. Following recovery, slices were placed at room temperature for 30min-1hr prior to recording. Cells were visualized by fluorescence to recognize fluorescent protein positive cells followed by infrared differential interference contrast microscopy for patching. Whole cell voltage-clamp recordings below the substantia gelatinosa were obtained under visual guidance using a 40x objective. Voltage-clamp recordings from retrogradely labeled PSDCs in laminae IV-V were obtained under visual guidance using a 40x objective. Patch electrodes (4-6 MΩ) were filled with a KCl-based internal solution containing 125mM KCl, 2.8mM NaCl, 2mM MgCl_2_, 2mM ATP-Mg^2+^, 0.3mM GTP-Na^+^, 0.6mM EGTA, and 10mM HEPES, and neurons were voltage clamped at −70mV. Action potential (AP) discharge patterns were studied in current-clamp. The membrane potential recorded ∼10 s after switching from voltage to current clamp was designated as the resting membrane potential (RMP) and subsequent recordings were made from this potential. AP discharge patterns were characterized by injecting a series of depolarizing step-currents (1.2 s duration, 5-10pA increments, delivered every 6 s, ranging from −80pA to 200pA) into the recorded neuron. AP discharge patterns were classified according to previously published criteria (Graham et al., 2004, Graham et al., 2007, Grudt and Perl, 2002, Yasaka et al., 2010). In brief, Initial Bursting (IB) neurons were characterized by AP discharge limited to the beginning of the depolarizing step; Delayed (D) firing neurons featured a prominent delay between the onset of the depolarizing step and AP discharge; Single Spiking (SS) neurons were characterized by the discharge of a single AP; Phasic (P) neurons were characterized by a burst of AP firing at rheobase (2-4 APs) that became persistent at steady state; Gap Firing (GF) neurons featured prominent gaps between AP at rheobase and/or steady state; and Reluctant (R) firing neurons did not discharge APs. Regular Spiking (RS) and Tonic (T) firing patterns were characterized by persistent AP discharge throughout the depolarizing and distinguished according to previously published criteria (Hughes et al., 2012). For dorsal root stimulation experiments, 300μm thick transverse spinal cord slices with dorsal roots attached were prepared as described above. Patch electrodes (2-4 MΩ) were filled with a CsCl-based internal solution containing 135mM CsMeSO_3_, 4mM ATP-Mg^2+^, 0.3mM GTP-Na^+^, 1mM EGTA, 3.3mM QX-314(Cl^-^ salt), 8mM Na_2_-Phoshocreatine and 10mM HEPES. Synaptic currents were evoked with electrical stimulation of dorsal roots using a suction electrode at Aβ fiber strength (≤ 25 μA, 20-100μs) (Nakatsuka et al., 2000, Torsney and MacDermott, 2006), and PSDC neurons were voltage-clamped alternatively at the reversal potential for synaptic inhibition and excitation to isolate excitatory postsynaptic currents (EPSCs) and disynaptic inhibitory postsynaptic currents (IPSCs), respectively. To activate ChR2 in acute slices, LED whole field illumination was used through a water immersion 40x objective. Aδ-LTMR axon terminals were stimulated with brief pulses (1-5ms) of blue light (473 nm, ∼5mW). Input resistance and access resistance were monitored continuously throughout each experiment and cells were excluded from analysis if these values changed by more than 10% during the experiment or if the resting membrane potential was higher than −50mV. Data were acquired using a Multiclamp amplifier, a Digidata 1440A acquisition system, and pClamp10 software (Molecular Devices). Sampling rate was 10 kHz, and data were low-pass filtered at 3 kHz. No correction for junction potential was applied.

#### Immunohistochemistry of free-floating sections

Male and female mice (P30-P35) were anesthetized with CO_2_ and perfused with 5-10mL modified Ames Media (Sigma) in 1x PBS, followed by 20-40 mL of 4% paraformaldehyde (PFA) in PBS at room temperature (RT). Vertebral columns (including spinal cords and dorsal root ganglia) were dissected from perfused mice and were post-fixed in 4% PFA at 4°C for 2-16 hr. Sagittal sections (50-150μm thick) of the lumbar spinal cord were cut on a vibrating blade microtome (Leica VT100S) and processed for immunohistochemistry (IHC) as described previously (Hughes et al., 2012). In brief, tissue samples were rinsed in 50% ethanol/water solution for 30 min to allow for enhanced antibody penetration. Three washes in a high salt Phosphate Buffer Saline (HS PBS) were conducted each lasting 10 min. The tissue was then incubated in a cocktail of primary antibodies in high salt Phosphate Buffer Saline containing 0.3% Triton X-100 (HS PBSt) for 48-72 hr at 4°C. Primary antibodies are listed in Key Resources Table. The tissue was washed in HS PBSt, then incubated in a secondary antibody solution in HS PBSt for 24 hr at 4°C. Secondary antibodies included an array of species-specific Alexa Fluor 405, 488, 546, and 647 conjugated IgGs (Invitrogen). The tissue was treated with another HS PBSt, prior to incubation in 4’,6-diamidino-2-phenylindole (DAPI) stain at a 1:5000 dilution. Tissue sections were then mounted on glass slides and coverslipped with Fluoromount Aqueous Mounting Medium (Sigma). The slides were stored at 4°C.

#### Spinal cord whole-mount immunohistochemistry

Male and female mice (P30-35) were anesthetized, perfused, and post-fixed as described above for whole-mount (WM) immunohistochemistry. The entire spinal cord with DRGs attached were dissected from the vertebral column, followed by fine dissection to remove dura and hemisect the spinal cord along the rostro-caudal plane. Tissue was then blocked in blocking solution (1% Triton X-100, 1% Tween-20, 5% normal goat serum in 1xPBS) for 4 hr, followed by incubation with primary antibodies diluted in blocking solution on a rocking platform for 2-3 days. Primary antibodies are listed in Key Resources Table. Spinal cords were then washed 6 × 1 hr in PBST (1% Triton X-100 in 1xPBS) and incubated in secondary antibodies diluted in blocking solution on a rocking platform for 2-3 days. Secondary antibodies included an array of species-specific Alexa Fluor 488, 546, and 647 conjugated IgGs (Invitrogen). Following this, cords were washed 6 × 1 hr in PBST and serial dehydrated in 50%, 75%, and 100% MeOH (2 hr each, and final overnight incubation in 100% MeOH). The next day, spinal cords were pinned to a glass dish coated using Sylgard 184 Silicone Elastomer Kit (Dow), cleared in BABB (BABB: 1 part Benzyl Alcohol: 2 parts Benzyl Benzoate) for 5 min, and mounted on slides using BABB as mounting medium. All steps were completed at room temperature.

#### Mouse crosses for overlap matrix analysis

The following mouse crosses were used to determine the % coverage of LTMR-RZ by the genetically labeled interneuron mouse lines (Figure S2D). For each cross at least three animals were analyzed with at least 100 GFP^+^ cells counted per animal. For tamoxifen regimens when CreER lines are used see Table S1B. For antibody species and dilution when immunohistochemistry is used see Key Resources Table. Excitatory matrix: *CCK*^*CreER*^*;*Igfbp5-GFP*; R26*^*LSL-Tom*^(Ai9). *CCK*^*CreER*^*;*5HTr6-GFP*;R26*^*LSL-Tom*^(Ai9). *CCK*^*CreER*^*;*Cbln2-GFP*; R26*^*LSL-Tom*^(Ai9). *CCK*^*CreER*^*;*PV-Tom*;R26*^*LSL-YFP*^(Ai3). *CCK*^*CreER*^*;*NeuroD4-GFP*;R26*^*LSL-Tom*^(Ai9). *CCK*^*CreER*^*; R26*^*LSL-Tom*^(Ai9) with PKCγ immunohistochemistry. 5HTr6-CreER*;*Cbln2-GFP*;R26*^*LSL-Tom*^(Ai9). 5HTr6-CreER*;*NeudoD4-GFP*;R26*^*LSL-Tom*^(Ai9). 5HTr6-CreER*;*Igfbp5-GFP*;R26*^*LSL-Tom*^(Ai9). 5HTr6-GFP with PKCγ and PV immunohistochemistry. NeuroD4-GFP with PKCγ and PV immunohistochemistry. Cbln2-GFP with PKCγ and PV immunohistochemistry. Igfbp5-GFP with PKCγ and PV immunohistochemistry. WT tissue with PKCγ and PV immunohistochemistry. Inhibitory matrix: Cdh3-GFP with PV immunohistochemistry. *Rorβ*^*GFP*^ with PV immunohistochemistry. Kcnip2-GFP with PV immunohistochemistry. Kcnip2-CreER*;*Cdh3-GFP*; R26*^*LSL-Tom*^(Ai9). *Rorβ*^*CreER*^*;*Kcnip2-GFP*;R26*^*LSL-Tom*^(Ai9). *Rorβ*^*CreER*^*;*Cdh3-GFP*;R26*^*LSL-Tom*^(Ai9).

#### Behavioral testing

Male mice of a mixed genetic backgrounds (C57BL/6J and FVB/NJ) were used for behavioral analyses. Testing was done beginning at 7 weeks of age, and in most cases, was completed by 12 weeks of age. All animals were group housed, with control and mutant animals in the same litters and cages. Littermates from the same genetic crosses were used as controls for each group, to control for variability in mouse strains/backgrounds. Animal numbers per group for behavioral tests are indicated in figures. All behavioral analyses were done by observers blinded to genotype.

For a detailed protocol of texture NORT, see (Orefice et al., 2016). In brief, for NORT assays mice were first habituated to an open field chamber by allowing free exploration of an empty chamber for 10 min for two consecutive days (day 1 and 2). Each of the two subsequent testing days involving texture NORT (day 3) and color/shape NORT (day 4) included two sessions. In the first session (learning phase), the mouse was placed in the testing arena with two identical objects placed in the center of the arena. Each mouse was allowed to explore the objects for 10 min. Animals were then removed from the testing arena and placed in a transport cage for 5 min. During this time, the arena was cleaned with 70% ethanol, and one of the objects was replaced with a novel object. The mouse was then placed back into the chamber and allowed to explore objects for 10 min (testing phase). The amount of time the mouse spent physically investigating (touching) each of the objects was assessed during both the learning and testing phases. If an animal did not physically touch both objects during the learning phase, it was excluded from NORT analysis. For textured NORT, textured objects (either smooth or rough) were 4 cm on each side and constructed of plexiglass. For color/shape NORT, wooden blocks that differed in shape, size and color were utilized. To avoid confounding whisker movements and sensations, whiskers were plucked three days prior to the start of habituation. The position of the mouse was tracked using custom MATLAB scripts. Whisking, nose pokes and investigation using the paws were all included in the time spent investigating objects, though for this assay over 90% of the time investigating objects is performed using the glabrous skin on paws (Orefice et al., 2016).

The response of mice to tactile and acoustic startle stimuli was measured using a San Diego Instruments startle reflex system (SR-LAB Startle Response System) (Orefice et al., 2016). In brief, for tactile PPI air puffs were administered to the back of the mouse to assess hairy skin sensitivity. A 1.5 PSI air puff prepulse stimulus strength was chosen because control animals of this particular Bl6/FVB mix showed a minimal, but statistically significant response to the stimulus alone, compared to baseline movement in the chamber without any stimulus (average response in controls, 8.19 ± 1.39%). Each mouse was placed in the chamber for a 5 min acclimation period, during which constant background noise of broadband white noise was presented. Background noise for the acoustic PPI testing sessions was 65 dB. Background noise for the tactile PPI testing sessions was increased to 75 dB, to ensure that that the animal could not hear the air puff prepulse. Acoustic PPI and tactile PPI sessions were run on separate days. For acoustic PPI, the prepulse was 20ms in duration and presented 100ms before the startle pulse (inter-stimulus interval, ISI). For tactile PPI, the prepulse intensity remained constant (1.5PSI, 50ms), and the ISI was varied from 50ms to 1 s in duration. Whole body flinch, or startle reflex, was quantitated using an accelerometer sensor measuring the amplitude of movement of the animal, within the cylindrical holder.

#### Neuronal reconstructions and morphometric analysis

Sagittal sections of lumbar spinal cord were immunostained as described above and z stack images were taken on a Zeiss LSM 700 confocal microscope using a 20X lens (Plan-Apochromat 20X/NA 0.8). Analysis was limited to the LTMR-RZ, using IB4 (IIiv border) as an upper limit and 250μm below IB4 as a lower limit. Confocal image stacks were loaded into the Neurolucida 360 software. Specific neurons from each image stack were reconstructed using the user-guided reconstruction tool. Reconstructions were saved and opened in Neurolucida Explorer software for morphological analysis. Basic information detailing somatic and dendritic measurements were retrieved from the reconstructions using Neurolucida software and graphed with GraphPad Prism. Sholl-based metrics detailed in Figure S3 including: Enclosing radius, Sum of Intersections, Critical Value (Nm), Critical Radius (Rc), Mean Value (Nav), Centroid Value, Centroid Radius, Ramification Index (RI), Regression Coefficient (k), Branching Index (BI), were obtained by analyzing intersection-based sholl data obtained in Neurolucida with MATLAB script written using previously described formulas (Ferreira et al., 2014, Garcia-Segura and Perez-Marquez, 2014, Rajković et al., 2016). The depth location within the LTMR-RZ was measured from the bottom of the IB4^+^ lamina IIiv using ImageJ software.

#### Linear Discriminant Analysis

We performed linear discriminant analysis (LDA) on 26 parameters collected from the neuronal morphometric analysis using the lda function in R, on a total of 200 excitatory and 137 inhibitory interneurons. These 26 parameters were chosen from a total of 46 metrics and deemed to be most important to interneuron classification due to their negative effect on classifier performance when removed from the parameter dataset. Prior to running LDA, all data was scaled and centered to adjust for differences in magnitude between metrics, and interneurons were randomly split into a training set (90%) and test set (10%). LDA using the training set was used to create a classifier, for which performance was assessed with the test set. The process of random splitting into training and test sets, followed by LDA and test set classification, was iterated 10,000 times while recording the incidence of true positives (TP), true negatives (TN), false positives (FP), and false negatives (FN) resulting from classification of the test set. These values were used to calculate classifier **precision** p = *TP / (TP+FP)*, **recall** *R = TP / (TP+FN)*, **fallout** *F = FP / (FP+TN)*, **miss** *R = FN / (FN+TP)*, and **accuracy** *A =* (*TP+TN) / (TP+TN+FP+FN)*.

To ask which categories of metrics were most important to classifier performance, we performed LDA and interneuron classification as described above while removing categories of variables relating to either cell location (Distance Below IB4), soma morphology (Enclosed Volume, Max Perimeter, Area, Feret Max Soma, Aspect Ratio, Roundness, Mean Area, Surface Area), dendritic spines (Spines, Spine Density), or dendritic morphology (Dendrite Quantity, Nodes, Length, Volume, Torsion Ratio, Convex Hull Volume, Convex Hull Area, Sum of Intersections, Critical Value, Critical Radius, Centroid Radius, Ramification Index, Regression Coefficient, Branching Index). The heatmap.2 function in R was used to construct a heatmap representing the reduction in classifier accuracy resulting from removal of these metrics.

#### Synaptic analysis

Within the LTMR-RZ, vesicular glutamate transporters (vGluTs) are well-established markers to label peripheral, local excitatory interneurons, and cortical pre-synaptic inputs, with differences in which vGluT subtype each population expresses. Established LTMR subtypes also display unique vGluT expression: C-LTMRs in the mouse express vGluT3 while Aβ- and Aδ-LTMRs express vGluT1 (Seal et al., 2009, Todd et al., 2003). Descending excitatory cortical projection neurons express vGluT1 while local excitatory interneurons express vGluT2 (Du Beau et al., 2012, Todd et al., 2003). Homer protein family members are expressed at postsynaptic densities (PSDs) of glutamatergic synapses where they play crucial roles in synaptic scaffolding and Ca^2+^ signaling. Importantly, Homer proteins are located further from the synaptic cleft (∼80nm) than other established markers of excitatory PSDs such as Shank proteins, PSD-95, or GluR1 subunits, making it possible to label these proteins without antigen retrieval (Dani et al., 2010, Gutierrez-Mecinas et al., 2016), and furthermore, Homer protein has been shown to be present at the majority of glutamatergic synapses within the dorsal horn (Gutierrez-Mecinas et al., 2016). Thus, in this study, Homer1 is used to detect the presence of all excitatory glutamatergic synapses, with the combined use of pre-synaptic markers, including vGluT1 and genetically expressed synaptophysin-tdTomato (via Ai34).

##### LTMR-RZ synaptic architecture analysis

Sagittal sections of lumbar spinal cord (50 μm) were immunostained as described above and z stack images were taken on a Zeiss LSM 700 confocal microscope using a 20X lens (Plan-Apochromat 20X/NA 0.8). Analysis was limited to the LTMR-RZ, using IB4 (lamina IIi-IIiv) as an upper limit and 250 μm below IB4 as a lower limit. Apposition analysis with Homer1^+^ and Ai34^+^ puncta was completed using published methods (Ippolito and Eroglu, 2010). For each animal used in analysis, a minimum of 5 sets of images, each image set comprising (2) 5 μm z stacks from a minimum of 3 separate sections, was used for analysis. Input analysis of synaptophysin-toTomato overlap with vGluT1 was analyzed using ImageJ software to isolate *Emx1*^*Cre*^*;*Ai34 or *Advillin*^*Cre*^*;*Ai34 puncta contained within vGluT1^+^ puncta; these puncta were subsequently counted using the Puncta Analyzer plugin. For each animal included in the analysis, a minimum of of 2 sets of images, each image set comprising (2) 3 μm z stacks, was used.

For determining the number of synapses per individual LTMR, as shown in Figure S1, whole mount staining was performed on perfused, post-fixed spinal cords from adult (P30-P35) mice. Tiled z stack images were taken on a Zeiss LSM 700 confocal microscope using a 20X lens (Plan-Apochromat 20X/NA 0.8) and used for subsequent analysis. For each image, ImageJ software was used to crop to a region of interest that contained only the central projection & synapses from a single neuron. These cropped images were blinded for subsequent analysis, in which the ImageJ Cell Counter plugin was used to count total synaptophysin-tdTomato^+^ puncta per neuron (based on a minimum size and intensity threshold). A minimum of 1 (for Aβ RA-LTMRs) or 3 (for C- and Aδ-LTMRs) cells were quantified per animal, with cells sampled across cervical, thoracic, and lumbar regions for all subtypes. Averages of these counts (n = 4 animals per LTMR subtype) yield the average number of synapses per neuron. To calculate total synaptic input from each LTMR population, the average number of synapses per C-, Aδ-, or Aβ RA-LTMR was multiplied by the relative abundance of these subtypes in the DRG, previously reported as 15%–20%, 7%, and 5% of total DRG neurons, respectively (Li et al., 2011, Luo et al., 2009, Rutlin et al., 2015). Further multiplication using an average of 10,000 neurons per mouse DRG (Gjerstad et al., 2002), and 62 DRGs (8 cervical, 13 thoracic, 6 lumbar, 4 sacral DRGs per side) completes the calculation to yield total synaptic input from each population (puncta per population = (puncta/neuron) × (% of DRG) × 62,000).

##### Distribution analysis of LTMR-RZ interneuron synapses

Sagittal sections of lumbar spinal cord (50 μm) were immunostained as described above and z stack images were taken on a Zeiss LSM 700 confocal microscope using a 20X lens (Plan-Apochromat 20X/NA 0.8). Low-level expression of synaptophysin-tdTomato in cellular cytosol was used to locate sparsely labeled cells and follow neurites to all tdTom^+^ synapses. ImageJ software and multipoint tool was used for marking synapses and exporting coordinates; center of cell soma and lamina IIiv border (using IB4 binding) were also marked and measured. Synaptic coordinates were then converted into their location in the dorsal-ventral axis relative to IB4. Cells with somas residing outside of the LTMR-RZ were not included in the analysis. A minimum total of 10 cells from 3 animals was used in this synaptic distribution analysis.

##### Analysis of pre- and post-synaptic inhibitory contacts from LTMR-RZ interneurons

Transverse sections of lumbar spinal cord (60 μm) were immunostained as described above and were scanned with a Zeiss LSM 710 confocal microscope equipped with argon multiline, 405 nm diode, 561 nm solid state, and 633 nm HeNe lasers, and a spectral detection system. Image stacks were obtained through a 63x oil-immersion lens (numerical aperture 1.4) and scanned at a z-separation of 0.3 μm. The resulting z stacks of were analyzed with Neurolucida for Confocal software (MBF Bioscience, Williston, VT). Laminar boundaries were determined by mapping the expression patterns of PV and vGluT1 (for laminae IIi and III), and overlaying templates of appropriate spinal segments obtained from the Allen Brain Atlas (http://www.brain-map.org/) onto projected images of immunolabeled sections.

For analyzing inhibitory contacts to myelinated afferents (*Advillin*^*Cre*^*;*Ai34) and descending corticospinal projections (*Emx1*^*Cre*^*;*Ai34) (n = 4 animals for each line), only channels corresponding to the reporter and vGluT1 were initially viewed and fifty boutons that contained either reporter and vGluT1 or only vGluT1, were selected randomly in each lamina. The channel corresponding to the vGAT labeling was then viewed. The proportion of terminals from either group that were contacted by vGAT terminals, and the mean number of vGAT contacts onto these boutons, was then determined.

For characterizing inhibitory inputs to vGluT1^+^ boutons in the LTMR-RZ from Cdh3^+^ (Cdh3-GFP*)*, PV^+^ (immunostaining), Rorβ^+^ (*Rorβ*^*CreER*^*;*Ai34), and Kcnip2^+^ (Kcnip2-CreER*;*Ai34) interneurons (n = 4 animals for each interneuron population), we first used Neurolucida for Confocal to randomly select fifty axon terminals per lamina that contained both the reporter and vGAT from confocal image stacks from each animal. The channel corresponding to vGluT1 labeling was then viewed, allowing us to determine the proportion of inhibitory terminals from each reporter line that target vGluT1 boutons in the LTMR zone. We then randomly selected fifty vGluT1 terminals from each lamina, before revealing the vGAT labeling followed by viewing the channel for the reporter. This allowed us to determine both the number of vGAT terminals in contact with each vGluT1 terminal, and the proportion of these terminals that expressed the reporter labeling. To determine the proportion of inhibitory reporter terminals that mediate postsynaptic (rather than presynaptic) inhibition in the LTMR recipient zone, a total of fifty reporter-expressing terminals that were also vGAT-immunoreactive were selected randomly (n = 3 animals for each interneuron population). Confocal image stacks were then analyzed using Neurolucida for Confocal to determine the proportion of inhibitory reporter terminals that apposed a gephyrin-immunoreactive punctum.

##### Array tomography

Anatomical synaptic contacts were validated using array tomography (AT) to confirm overlap of synapses from primary sensory neurons (*Advillin*^*FlpO*^*;R26*^*FSit*^) with synaptic markers used in this study as well as other known synaptic markers. This procedure was completed by the Harvard Neurobiology Imaging Facility and analysis was conducted as previously published (Saunders et al., 2015). Mice used for this analysis were perfused as described above; lumbar spinal cord samples were post-fixed at 4°C overnight, rinsed 3 × 30min in 1xPBS, and sectioned into 150 μm slabs using a vibratome (Leica VT100S). Lumbar spinal cord was then dehydrated, embedded in LR white resin and serially sectioned at 70nm using an ultramicrotome (Saunders et al., 2015). After embedding and before sectioning, dorsal and ventral horns of the spinal cord were visually identified by morphological differences, and ventral horns were trimmed from block to ensure the appropriate region of the spinal cord was isolated for subsequent imaging and analysis. Staining, imaging, and post-imaging alignment and background subtraction was performed as previously described (Saunders et al., 2015); see Key Resources Table for antibodies used. DAPI and GFP staining were used to determine regions of interest within the LTMR-RZ on each section. Four images were then acquired and stitched into a final image; DAPI staining across each staining session was used to align images across imaging sessions. Image analyses were carried out using previously written MATLAB scripts provided by the lab of Dr. Bernardo Sabatini (Saunders et al., 2015). GFP volumes (defined by spanning multiple planes with minimum size and brightness thresholds) and synaptic markers (defined by minimum size and brightness thresholds) were computationally detected from image stacks, excluding DAPI nuclei and regions lacking tissue. Colocalization analyses of GFP and synaptic markers was performed within (distance = 0) and at varying distances outside (102-512nm) of GFP volumes. Mean occurrence of colocalization per voxel was compared to that of 1,000 rounds of randomized immunopunctae. Z scores were calculated for distance = 0 as follows: [μ_actual_-μ_randomized_]/σ_randomized_ where μ is mean occurrence of colocalization per voxel and σ is standard deviation. A total of n = 3 animals with 2 stacks each (each stack comprising 25-35 70nm sections) was used for this analysis.

##### Input and connectivity analysis

Synaptic input and connectivity analysis (as presented in Figures 6 and 7) was performed on mice in which LTMR-RZ interneuron BAC-GFP transgenic lines were crossed with Cre and CreER lines of input populations of interest and the synaptophysin-tdTomato reporter line (Ai34). Thus, in a single animal, one LTMR-RZ interneuron population (Cbln2^+^, NeuroD4^+^, CCK^+^, 5Htr6^+^, Igfbp5^+^, Kcnip2^+^, Rorβ^+^, or Cdh3^+^) along with the pre-synaptic boutons of one input population (descending corticospinal projections, Aβ RA-LTMRs, Aδ-LTMRs, or C-LTMRs) were genetically labeled. Immunostaining allowed for the additional detection of PKCγ^+^ and PV^+^ interneuron populations in GFP-negative animals (PVe and PVi populations were distinguished by morphology), as well as pre- and post-synaptic markers used in the analysis (primarily vGlut1 and Homer1). All animals used in this analysis were perfused, postfixed, sectioned (50 μm, lumbar spinal cord), and immunostained as described above.

Z stack images of spinal cord slices were taken on a Zeiss LSM 700 confocal microscope using a 40X oil-immersion lens (Zeiss; Plan-Apochromat 40X/NA 1.40) and scanned at a z-separation of 0.5 μm. Images were taken in lamina IIiv-IV of the dorsal horn, which was defined as between the lamina IIiv border (marked by IB4 binding) and 250 μm below that border. Bias to particular regions of the LTMR-RZ (particularly in the dorsal-ventral axis) based on input population was actively avoided by not observing the input population channel until a particular interneuron cell was selected for imaging. Further, for interneuron populations spanning multiple laminae, cells were selected and imaged in a repeating order of dorsal to ventral, ensuring that dorsal and ventral components of the population were sampled for analysis. Imaging parameters (laser power, gain/offset, averaging, dwell time, etc.) were consistent across each stain on all animals. For example, Homer1 (Alexa Fluor 647 secondary) was imaged using the same parameters in *all* animals; whereas GFP (Alexa Fluor 488 secondary) was imaged using the same parameters for all Igfbp5-GFP animals, but would differ from that of the other BAC-GFP transgenic lines.

All images were first prepared for analysis using ImageJ: using the channel of interneuron labeling, two masks were generated - one using a standardized threshold for signal in this channel and a second by expanding this first mask by 1 μm in all dimensions. These masks were then used to isolate pre- and post-synaptic labeling by multiplying these channels (using the Image Calculator function) with the expanded and non-expanded masks, respectively. Thus, when recombined for analysis, each image contained pre- and post-synaptic labeling that was restricted to sites of expanded or non-expanded GFP overlap, respectively (see Figure S6B). Blinded images were then analyzed for these inputs by eye, using the Cell Counter ImageJ plugin. Anatomical excitatory inputs were identified using Homer1 antibody overlap with the labeled interneurons of interest; each Homer1^+^ puncta represents one anatomical excitatory input. These excitatory inputs were defined as originating from an input population of interest when the pre-synaptic marker of that population (vGluT1 or synaptophysin-tdTomato) partially (minimum of 10% Homer1^+^ pixels overlapping with input pixels) or fully overlapped with a Homer1^+^ puncta. All analysis was restricted to neurons where the cell body was clearly in view and associated with the respective neurite. Puncta were counted as a factor of location: cell body, proximal neurite (within the first 50 μm) or distal neurite (beyond the first 50 μm). For each genotype, a minimum of 3 animals was used for analysis, with a minimum sampling from each animal of 4 neurons per cellular compartment (minimum total length of 500 μm and 50 μm analyzed for proximal and distal neurites, respectively).

For broad and LTMR subtype-specific connectivity profiles of each LTMR-RZ interneuron subtype (as presented in Figure 6B and 7A), synaptic input ratios were calculated as follows. From each cell, if multiple neurites were analyzed, the synaptic counts and neurite lengths were summed (keeping proximal and distal separate), and puncta per length (μm) and puncta per surface area (μm^2^) were calculated for neurites and somas, respectively. Homer1^+^ puncta represent total excitatory input to the cell, and so to obtain the proportion (%) of this excitatory input from a particular input population, Homer1^+^Ai34^+^ puncta per μm^2^ were divided by total Homer1^+^ puncta per μm^2^. These values were subsequently divided by the input population normalization value to account for variability in labeling efficiency (see next paragraph). For each animal, these normalized values for proportion of excitatory input from each cellular compartment were then averaged across all neurons; these were then averaged to obtain the final normalized average ± SEM (n = animal number) proportion of excitatory inputs. Thus, proportion (%) of excitatory input from input population ‘A’ = [(total # A^+^homer1^+^ puncta/μm) ÷ (total homer1^+^ puncta/μm)] ÷ normalization value (this calculation is done separately for each cellular compartment). Subtractive calculations (such as those used in Figure 6B) utilized these final averages across all animals of a particular genotype. For comparisons made between input populations, the normalized average puncta per μm for each input was totalled across all 11 interneuron lines, and the puncta per μm of inputs onto each interneuron subpopulation was divided by this total. The result of this computation is to show, of the anatomical inputs *onto these 11 interneuron populations*, what proportion is dedicated to each interneuron subtype.

##### Normalization value

The reliance on tamoxifen treatment for recombination and expression of synaptophysin-tdTomato in our LTMR-CreER lines presents the likelihood of variable labeling from animal-to-animal. Thus, for all animals analyzed, the average synaptophysin-tdTomato puncta per image area (μm^2^) was calculated. Unprocessed images (the same as those used for the connectivity profile counts) were used to isolate synaptophysin-tdTomato puncta in a particular region of interest (the lamina-specific innervation target of that population) with a standardized threshold and then count total puncta number using the ImageJ Puncta Analyzer program. For each animal, a minimum of puncta counts from 3 images was obtained and averaged. These values were compared across all animals of a single Cre or CreER line, and the maximum synaptophysin-tdTomato puncta per μm^2^ was determined. For each animal, the average puncta per μm^2^ is then divided by this maximum value to determine the normalization value for labeling efficiency, which is subsequently used as described above. This was also completed for vGluT1 staining to account for differences in staining efficiency and to optimize subtractive calculations. Normalization value for input population ‘A’ = (total # A^+^ puncta) ÷ (total area in ROI).

### Quantification and Statistical Analysis

All data are expressed as the mean ± the standard error of the mean (SEM), unless otherwise stated in the figure legend.

For morphological/physiological comparisons a Student’s t test was used to compare excitatory and inhibitory cohorts. One-way ANOVAs are expressed as an F-statistic and P value within brackets, and post hoc comparisons were performed using the post hoc test indicated in the figure legend. The p values of post hoc comparisons between groups are represented with asterisks above brackets over the indicated groups using a bracketed line in the figures.

For behavior, the number of animals per group used in each experiment is denoted within the bar for that group in each panel. Unless otherwise stated, a Student’s t test was used to compare a group to chance performance (0% for NORT), or to compare mutants to their control littermates. If significant differences between mutants and control littermates were observed, this was indicated by an asterisk over the indicated groups. Main effects of genotype to tactile PPI from one-way ANOVAs are expressed as an F-statistic and P value within brackets.

For LTMR-specific connectivity profiles, each input population was compared to a hypothetical mean (0%) using a one-sample t test. If these values were not statistically significantly (p > 0.05) above 0%, lack of significance was indicated by “n.s.” above the respective bar graph. Comparisons between input populations onto a single interneuron population were performed using one-way ANOVA, and main effects from one-way ANOVAs are expressed as an F-statistic and P value within brackets. Post hoc comparisons were performed using Tukey’s test. The p values of post hoc comparisons between groups are represented with asterisks above brackets over the indicated groups using a bracketed line in the figures.

## Author Contributions

V.E.A., E.D.K., and D.D.G. conceived the study. V.E.A. and E.D.K. performed experiments with help from A.M.C., M.W.S., A.A.T., L.L.O., K.A.B., L.B., B.J.S., K.A.B., J.Z., C.T., J.H., and D.I.H. V.E.A. generated *PKCγ*^*mGFP*^, *PKCγ*^*CreER*^, and Cdx2-NSE-FlpO lines; V.E.A., M.R., and S.B.N. generated the Rorβ^CreER^ line; and A.L.Z. and V.E.A. generated the *TH*^*2A−CreER*^ mouse line. M.W. provided antibodies. L.K. and N.H. generated the Cdh3, 5HTr6, Kcnip2, and BAC-CreER mouse lines. V.N. and S.M.D. generated the *RC*::*PFtox* (*R26*^*PFtox*^) and *RC*::*FPSit* (*R26*^*FPSit*^) mouse lines. M.W.S. did LDA analysis, with help from T.G.O. V.E.A., E.D.K., and D.D.G. wrote the manuscript, with input from all authors.

## Supplemental Information

Table S1. Mouse Lines Generated and Characterized, Related to Figure 2 (A) A summary of fluorescent reporter mouse lines. (B) A summary of recombinase reporter mouse lines.
